# *Angiotensin*-*converting enzyme Ance* is cooperatively regulated by Mad and Pannier in *Drosophila* imaginal discs

**DOI:** 10.1038/s41598-017-13487-w

**Published:** 2017-10-13

**Authors:** Ah-Ram Kim, Eun-Bee Choi, Mi-Young Kim, Kwang-Wook Choi

**Affiliations:** Department of Biological Sciences, Korea Advanced Institute of Science and Technology (KAIST), Daejeon, 305-701 Republic of Korea

## Abstract

Angiotensin-converting enzyme (ACE) is an evolutionarily conserved peptidyl dipeptidase. Mammalian ACE converts angiotensin I to the active vasoconstrictor angiotensin II, thus playing a critical role for homeostasis of the renin-angiotensin system. In *Drosophila*, the ACE homolog Ance is expressed in specific regions of developing organs, but its regulatory mechanism has not been identified. Here we provide evidence that *Ance* expression is regulated by a combination of Mad and Pannier (Pnr) in imaginal discs. We demonstrate that *Ance* expression in eye and wing discs depends on Dpp signaling. The Mad binding site of *Ance* regulatory region is essential for *Ance* expression. *Ance* expression in imaginal discs is also regulated by the GATA family transcription factor Pnr. Pnr directly regulates *Ance* expression by binding to a GATA site of *Ance* enhancer. In addition, Pnr and Mad physically and genetically interact. *Ance* null mutants are morphologically normal but show genetic interaction with *dpp* mutants. Furthermore, we show that human SMAD2 and GATA4 physically interact and *ACE* expression in HEK293 cells is regulated by SMAD2 and GATA4. Taken together, this study reveals a cooperative mechanism of *Ance* regulation by Mad and Pnr. Our data also suggest a conserved transcriptional regulation of human *ACE*.

## Introduction

Angiotensin converting enzyme (ACE) is a zinc-dependent dipeptidyl carboxypeptidase that converts Angiotensin I to Angiotensin II. In mammals, ACE plays important roles in the regulation of the renin-angiotensin system (RAS) on blood pressure homeostasis by generating Angiotensin II, an active vasoconstrictor that increases blood pressure. ACE has also been implicated in other biological functions including immune response, cytokine expression, and cell proliferation^1–4^. *ACE* knockout mice show not only hypotension but also renal developmental abnormalities, indicating a complex role of ACE^5,6^. However, the transcriptional regulation and function of ACE in developing tissues *in vivo* is not well understood.

Ance is a *Drosophila* homolog of the mammalian Angiotensin-converting enzyme (ACE). Due to the extensive glycosylation of human ACE, *Drosophila* Ance has been extensively used for three dimensional structure analysis as a model for studying ACE inhibitor binding^7–9^. Despite the evolutionary conservation of Ance, *Drosophila* does not have angiotensin homologs, raising questions on the biological function of Ance. Interestingly, *Ance* expression is developmentally regulated by Decapentaplegic (Dpp) signaling during *Drosophila* embryogenesis^10,11^. Dpp is the *Drosophila* homolog of the vertebrate bone morphogenetic proteins (BMPs) and the transforming growth factor beta (TGFβ) that acts as a signaling factor for diverse morphogenetic events. The Dpp signal is mediated by receptor serine/threonine kinases, such as Thickveins (Tkv), Saxophone (Sax), and Punt. The activated receptors induce phosphorylation of the Mad transcription factor leading to activation of downstream target genes^12–14^.

Secreted Dpp forms an extracellular concentration gradient to induce graded responses in cells receiving the signal^15–17^. In embryogenesis, a gradient of Dpp from the dorsal midline leads to differential transcriptional activation in the dorsal ectoderm according to the signal threshold. The highest level of Dpp activity induces *Ance* expression in the presumptive amnioserosa through Mad binding to the *Ance* enhancer^18,19^. On the contrary, lower levels of Dpp signaling lead to the expression of the GATA family transcription factor *pannier (pnr)* ^19,20^. Dpp signaling also plays a key role in the patterning and growth of imaginal discs to generate adult organs. For example, Dpp is critical for the progression of the morphogenetic furrow in the eye imaginal disc^21,22^ and for wing disc growth signaling from the anterior-posterior boundary^15,23^. Dpp signaling is also required for wing venation during pupal development^24–26^. In eye discs, Pnr is known to be involved in establishment of the dorsoventral (DV) boundary by regulating the dorsal-specific gene expression^27^, although the relationship with Dpp signaling is unknown. As in embryogenesis, Dpp signaling affects *pnr* expression in wing discs. The dorsal region of the wing disc is a primordium for the notum, the dorsal part of the thorax. Dpp signaling in this region regulates Wg expression by inducing *pnr* ^28,29^. Interestingly, previous studies have shown that *Ance* is specifically expressed in the dorsal region of the eye disc and the notum region of the wing disc^30^. We have noticed that the sites of *Ance* expression in these discs may correspond to the territories of *pnr* expression in the eye disc. These observations raise questions about whether the *Ance* expression in these discs is related to the Dpp signaling and the Pnr function.

Despite the region-specific patterns of *Ance* expression in imaginal discs, little is known about how *Ance* expression is regulated and what role Ance plays in development. It has been reported that male accessory glands secrete Ance proteins^31,32^ and that males homozygous for *Ance* hypomorphic alleles are infertile due to defective spermiogenesis^33^. Moreover, some of the *Ance* mutant alleles are lethal^10^, indicating the requirement of Ance for development. Unexpectedly, new deletion *Ance* alleles generated in this work are fully fertile and viable, indicating that Ance is not critically required under normal condition. However, it is possible that Ance may become important when an interacting pathway is compromised. Thus, we have addressed two main issues in this study: the regulation of *Ance* expression and the possible genetic interactions between *Ance* and its regulators. We also asked if the mechanism for *Ance* gene regulation is shared in human cells.

Here we demonstrate that *Ance* expression in the eye and wing discs is regulated by Dpp signaling through Mad binding to the *Ance* regulatory region. *Ance* expression in these discs also depends on Pnr binding to one of the GATA sites in the *Ance* regulatory region. Furthermore, Mad and Pnr show physical interaction. *Ance* null mutants are fertile and morphologically normal. However, the *Ance* mutations show genetic interaction with *dpp* mutants. We also show that human *ACE* gene expression is regulated by SMAD2 and GATA4 transcription factors. Taken together, *Ance* is regulated by a combination of Dpp and Pnr and is involved in modulating some of Mad functions, providing insights into the regulation and function of mammalian ACE.

## Results

### Expression patterns of *Ance*-*lacZ* and Ance protein in eye and wing discs

The imaginal disc is a sac-like structure consisting of two epithelial layers, a disc proper (DP) and a peripodial epithelia (PE). While the disc proper is a columnar epithelium, peripodial cells form a thin squamous epithelium. *Ance* mRNA expression was shown to be enriched in the dorsal region of eye and wing discs^30^. We have found that this pattern of *Ance* mRNA is similar to that of *pnr* expression. Since *pnr* is selectively expressed in the peripodial cells of eye discs^34,35^, we investigated whether *Ance* is also preferentially expressed in the peripodial epithelium.

**Figure 1:**
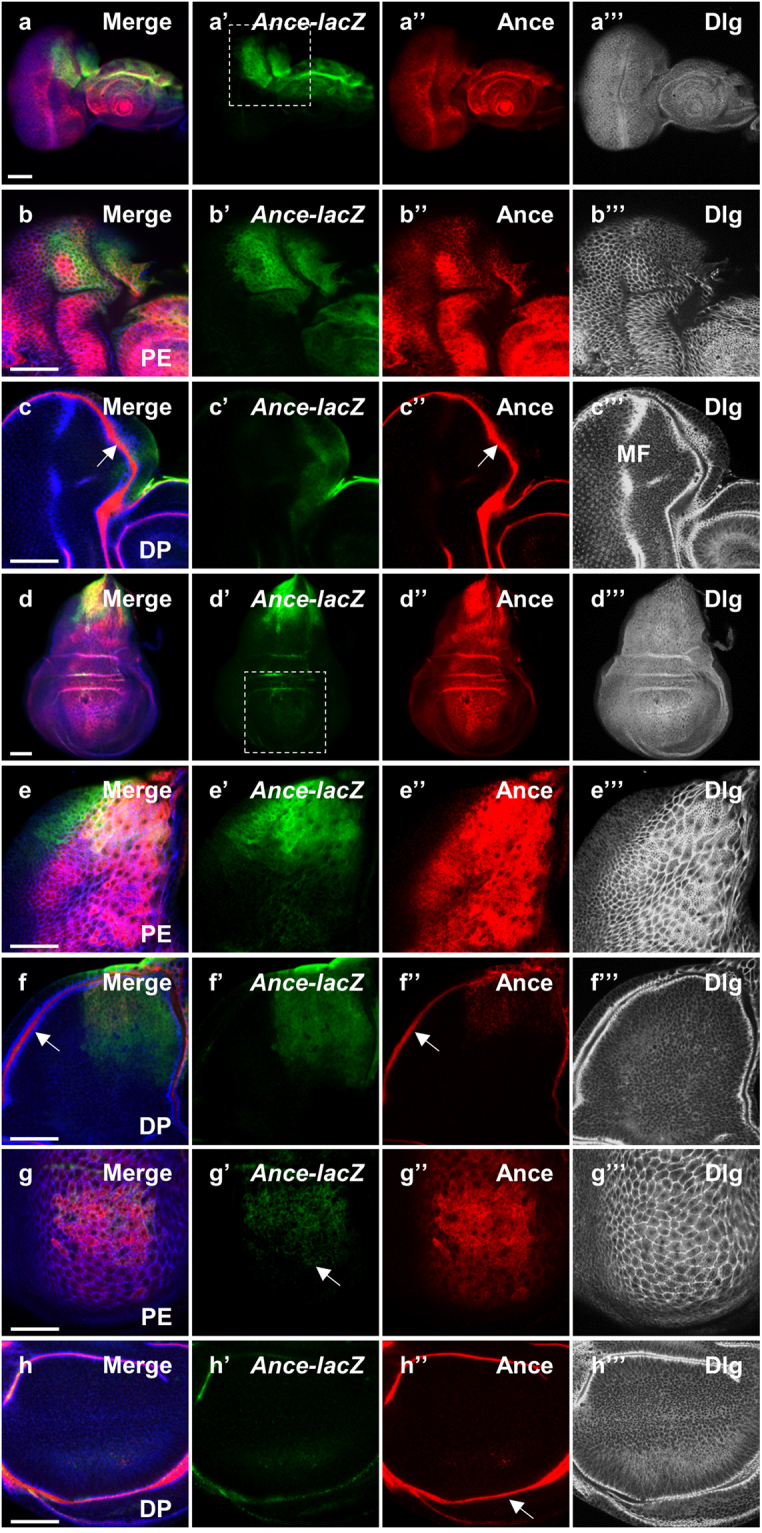
Expression pattern of *Ance*-*lacZ* and Ance protein in eye and wing discs. (**a**–**c**) Immunostaing of eye discs for *Ance*-*lacZ* (green), Ance (red) and Dlg (blue channel in black/white). (**a**-a”’) Low magnification views of an eye-antenna disc sectioned at the peripodial epithelium (PE) level. (a) Merge. (a’) *Ance*-*lacZ*. (a”) Ance protein. (a”’) Dlg. (**b**-b”’) High magnification views of a dorsal half of the eye disc shown in (a-a”’). (b) Merge. (b’) *Ance*-*lacZ* expressed in the dorsal PE. (b”) Ance protein. (b”’) Dlg staining of PE cells. (**c**-c”’) Dorsal region of the eye disc sectioned at the disc proper (DP) level. (c) Merge. (c’) *Ance*-*lacZ* is weakly expressed in the anterior dorsal region of DP. (c”) Ance protein in the lumen between PE and DP in the disc margin (arrow). (c”’) Dlg staining shows differentiating retinal cells posterior to the morphogenetic furrow (MF). It also shows a cross-section view of the lumenal space between the PE and DP layers in the disc margin. (**d**–**f**) Immunostaining of wing discs. (**d**-d”’) Low magnification views of a wing disc sectioned at the PE level. (d) Merge. (d’) *Ance*-*lacZ* strongly expressed in the dorsal region. (d”) Ance protein found in most PE cells. (d”’) Dlg staining of PE cells. (**e**-e”’) High magnification views of dorsal half of the wing disc shown in (d-d”’). (e’) *Ance*-*lacZ* expression in dorsal PE cells. (e”) Ance protein. (e”’) Dlg staining of dorsal PE cells. (**f**-f”’) Dorsal region of the wing disc sectioned at the DP level. (f’) *Ance*-*lacZ* expression is weakly detected in the dorsal region of DP. (f”) Ance protein in the lumen (arrow). (f”’) Dlg staining shows DP cells and a cross-section view of the lumen between the PE and DP layers in the disc margin. (g-g”’) High magnification views of the wing pouch region at the PE level shown in (d-d”’). (g’) *Ance*-*lacZ* expression (arrow). (g”) Ance protein. (g”’) Dlg staining of dorsal PE cells. (**h**-h”’) High magnification views of the wing pouch region at the DP level shown in (d-d”’). (h’) No *Ance*-*lacZ* expression in the pouch. (h”) Ance protein localized in the disc lumen (arrow). (h”’) Dlg staining of DP cells. Scale bar, 50 μm (a-h”’).

We utilized an *Ance*-*lacZ* reporter that reflects the dorsal expression of *Ance* mRNA in embryos^11^. First, we found that *Ance*-*lacZ* was dorsally expressed in eye and antenna discs (Fig. 1a,b), which resembled the pattern of *Ance* mRNA expression shown in a previous report^30^. Apical confocal sectioning of eye discs indicated that *Ance*-*lacZ* expression was preferentially localized to the peripodial epithelium (Fig. 1a’,b’) like that of *pnr* ^34,35^. Immunostaining with membrane marker anti-Discs-large (Dlg) antibody showed that *Ance*-*lacZ* expressing cells are large peripodial cells (Fig. 1a”’, b”’). We also checked the pattern of Ance protein expression using an anti-Ance polyclonal antibody^36^. Ance protein was found in most peripodial cells of the eye disc in contrast to the dorsally localized *Ance*-*lacZ* reporter expression (Fig. 1a”, b”).

In more basal sections, Dlg was detected in an array of differentiating photoreceptor cells in the disc proper (Fig. 1c”’) where *Ance*-*lacZ* was weakly detected in the anterior dorsal region (Fig. 1c’). Tangential sectioning at the disc proper level also shows a cross-section view along the margin of the disc. In this marginal area, Ance was highly localized in the lumenal space between the peripodial epithelium and the disc proper (Fig. 1c, c”, c”’). Although Ance protein was not clearly detected in the differentiating retina posterior to the morphogenetic furrow (Fig. 1c”), closer examination revealed weak levels of Ance in photoreceptor cells (arrowheads in Fig. S1e). Since *Ance*-*lacZ* is preferentially expressed in the dorsal peripodial cells, it can be inferred that the Ance protein detected widely in other areas of the eye disc probably originates from the secretion from cells expressing Ance.

Next, we examined the *Ance* expression in the wing disc. *Ance*-*lacZ* expression was abundant in the peripodial cells of the notum area (Fig. 1d–e”’), which is similar to the *pnr* expression pattern^27,34,35^. Weak expression of *Ance*-*lacZ* was also detected in the notum disc proper cells (Fig. 1f’). In contrast to the high levels of *Ance*-*lacZ* expression in the notum region, *Ance*-*lacZ* was weakly detected in the peripodial cells that cover the wing pouch region (Fig. 1g’). Ance protein in the wing disc was detected more broadly than the region of *Ance*-*lacZ* expression (Fig. 1d,e,g). Ance was abundant in the lumenal space (Fig. 1f”, h”), thus in contact with most disc proper cells including the notum and the wing pouch (Fig. S2). Taken together, the expression patterns of *Ance*-*lacZ* and Ance protein suggest that *Ance* mRNA is highly expressed in the dorsal peripodial region, while Ance protein is secreted to neighboring peripodial cells, lumenal space, and most disc proper cells.

### *Ance* expression is regulated by Dpp signaling during larval development

**Figure 2:**
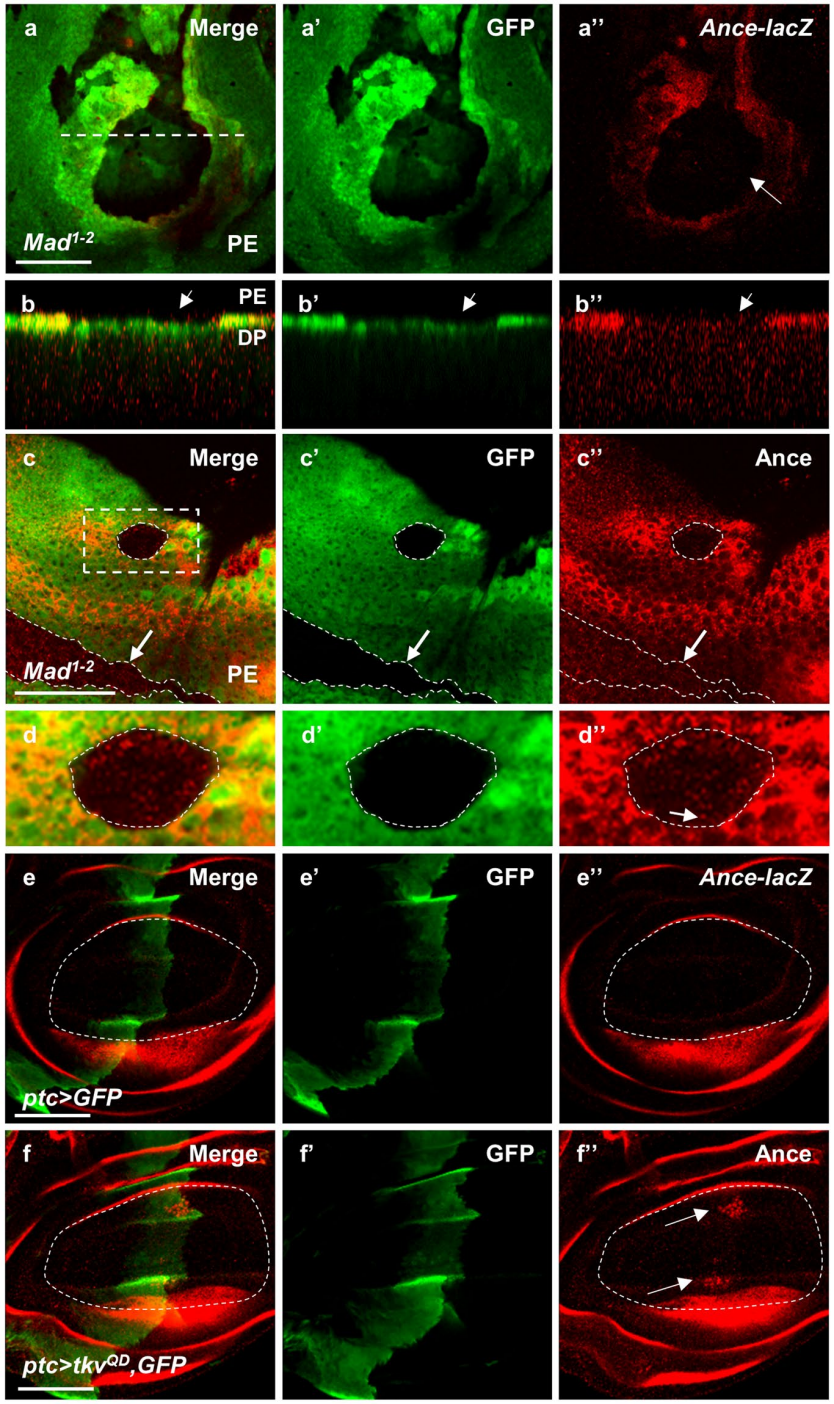
Dpp signaling is required for *Ance* expression in eye and wing discs. (**a**,**b**) Wing disc containing *Mad* ^*1*-*2*^ mutant clones stained for GFP clone marker and *Ance*-*lacZ*. Large *Mad* ^*1*-*2*^ mutant clones (GFP-negative) are shown in the wing pouch region (the box area in Fig. 1d’). *Ance*-*lacZ* expression is nearly absent in *Mad* ^*1*-*2*^ clones (arrow). (a) Merge of (a’-a”) at the PE level. (a’) GFP. (a”) *Ance*-*lacZ*. (**b**-b”) Z-section view of the dashed line in (a). Loss of *Ance*-*lacZ* expression in *Mad* ^*1*-*2*^ clone is indicated by the arrow. (**c**,**d**) Eye disc containing *Mad* ^*1*-*2*^ mutant clones stained for GFP clone marker and Ance protein. (c) Merge of (c’-c”) at the EP level. (c’) GFP. (c”) Ance protein. *Mad* ^*1*-*2*^ clone located away from the *Ance*-*lacZ* expressing area (marked by arrow) shows normal level of Ance protein. A *Mad* ^*1*-*2*^ mutant clone in the dorsal PE region (the box area in Fig. 1a’) shows a low level of Ance staining (dotted circle). (**d**-d”) High magnification views of the box area in (c). A low level of Ance protein can be detected (Arrow). (**e**-e”) Wing disc with GFP overexpression by *ptc*-*Gal4*. (e) Merge of an apical section images (e’-e”). (e’) GFP in the *ptc*-expressing region. (e”) No ectopic Ance expression in the *ptc* area of the wing pouch (circle). (**f**–f”) Wing disc with *tkv* ^*QD*^ overexpression by *ptc*-*Gal4*. (f) Merge of apical section images (f’-f”). (f’) GFP in the *ptc*-expressing region. (f”) Ectopic Ance expression in the proximal sites (arrows) in the *ptc* area of the wing pouch. Scale bars, 50μm (a–f”).

*Ance* expression is known to be regulated by Dpp signaling during embryogenesis^10,11^. Because Dpp signaling can activate different transcriptional target genes in different tissues and stages^18,19,37^, it is important to check whether Dpp signaling is required for the specific pattern of *Ance* expression in larval imaginal discs. We therefore examined the effects of loss of *Mad*, the transcription factor that mediates Dpp signaling, on *Ance*-*lacZ* expression. *Ance*-*lacZ* reporter expression was nearly undetectable in *Mad* mutant clones generated in the peripodial cells of wing discs (Fig. 2a,b and S3a). In addition, loss of Tkv, the receptor serine/threonine kinase for the Dpp ligand, resulted in reduced *Ance*-*lacZ* reporter expression in peripodial cells of eye disc (Fig. S4a,b).

We also tested the effects of loss of *Mad* on Ance protein expression. When *Mad* ^*1-2*^ mutant clones were generated in the regions distant from the dorsal domain of *Ance*-*lacZ* expression in eye disc, they showed normal level of Ance protein (arrow in Fig. 2c). This result suggests that Ance protein in these clones is provided non-autonomously from the *Mad* ^+^ *Ance*-expressing cells. *Mad* ^*1-2*^ clones located within the *Ance*-expressing region showed Ance proteins within the clones but at a reduced level (Fig. 2c,d).

We also checked whether ectopic Dpp signaling can activate Ance expression using Gal4-UAS system^38^. When *GFP* was overexpressed along the anterior-posterior boundary of wing disc using *ptc*-*Gal4* driver (labelled as *ptc* > *GFP*), there was no ectopic Ance expression in the *ptc*-expressing area (Figs 2e and S5a). However, overexpression of *tkv* ^*QD*^, a constitutively active form of *tkv*, led to ectopic expression of Ance in the *ptc* region of wing disc (Figs 2f and S5b). Interestingly, the ectopic Ance expression was mainly localized to two proximal spots in the *ptc* region of the wing pouch (Fig. S5b), suggesting that Dpp signaling can activate Ance expression in specific regions. Together, these results suggest that Dpp signaling is necessary and sufficient for *Ance* expression in specific territories of imaginal discs.

### *Ance* is dispensable for development

**Figure 3:**
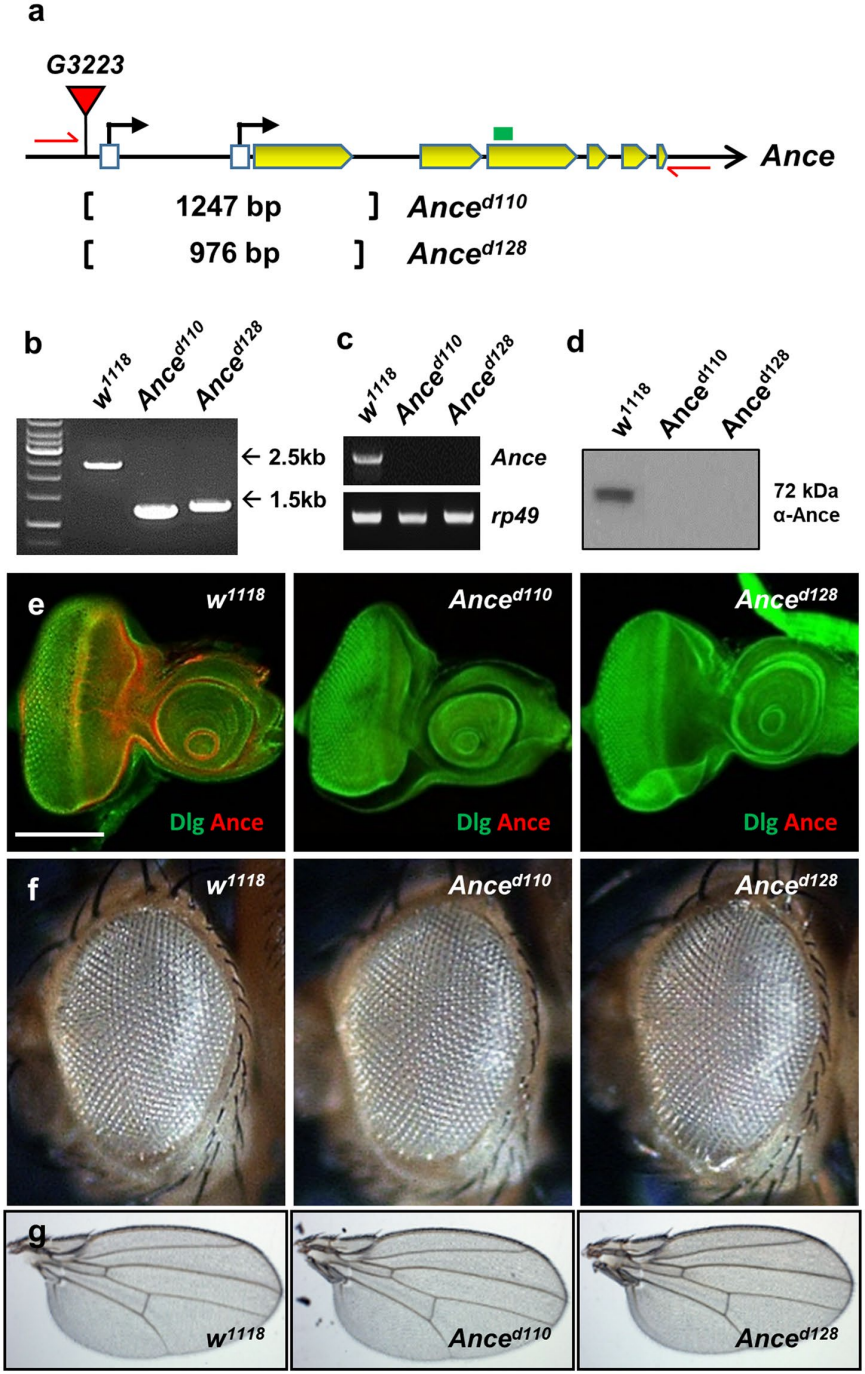
*Ance*-deletion mutants are viable. (**a**) Schematic structure of the *Ance* gene. The *G3223* line has a P-element inserted in the 5′ UTR of *Ance*. *Ance* ^*d110*^ and *Ance* ^*d128*^ mutants were generated by imprecise excision of *G3223*. Two mutants lack the first exon of *Ance* gene. Red arrows and green box indicate genomic PCR primers used in (b) and RT-PCR in (c), respectively. (**b**) Confirmation of deletions in *Ance* mutants. Genomic PCR results of *w* ^*1118*^, *Ance* ^*d110*^, and *Ance* ^*d128*^ show smaller PCR products in two *Ance* mutants compared to *w* ^*1118*^ control. (**c**) RT-PCR results of *w* ^*1118*^, *Ance* ^*d110*^, and *Ance* ^*d128*^. No PCR product is detected in two *Ance* mutants. (**d**) Ance protein is not detected in *Ance* ^*d110*^ and *Ance* ^*d128*^ mutants. (**e**) Absence of Ance staining in *Ance* mutants. Eye discs of *w* ^*1118*^, *Ance* ^*d110*^, and *Ance* ^*d128*^ stained for Ance and Dlg. Scale bar, 100 μm. (**f**) Adult eye morphology of *w* ^1118^, *Ance* ^*d110*^, and *Ance* ^*d128*^. *w* ^*1118*^ control shows the normal eye. *Ance* ^*d110*^ and *Ance* ^*d128*^ show normal eye morphology. (**g**) Adult wing morphology of *w* ^*1118*^, *Ance* ^*d110*^, and *Ance* ^*d128*^. *w* ^*1118*^ control shows the normal wing. *Ance* ^*d110*^ and *Ance* ^*d128*^ show normal wing morphology.

Since *Ance* is expressed in specific domains of discs, we tested whether Ance plays a role in the development of these organs. To examine the function of Ance, we generated two *Ance*-deletion mutants, *Ance* ^*d110*^ and *Ance* ^*d128*^, by imprecise excision of a P-element inserted in 5′ UTR of the *Ance* gene. In both mutants, the first exon and adjacent regions were deleted (1247 and 976 bp deletion in *Ance* ^*d110*^ and *Ance* ^*d128*^, respectively), which was confirmed by genomic PCR and sequencing (Fig. 3a,b). *Ance* mRNA and proteins were not detected in these deletion mutants, which were checked by RT-PCR (Fig. 3c), western blot (Fig. 3d), and immunostaining of imaginal discs (Fig. 3e), indicating that these two mutations are null alleles. These *Ance*-deletion mutants were viable and fertile with no obvious morphological defects in adult eyes and wings (Fig. 3f,g). This was unexpected because it was previously reported that *Ance* mutants show lethality or infertility^10,33^. Interestingly, *Ance* null mutants generated in this work fully complemented the *Ance* mutants (*Ance* ^*34Eb–1*^ and *Ance* ^*34Eb–2*^) described in earlier reports (data not shown). In contrast, the lethality of *Ance* ^*34Eb–1*^ and *Ance* ^*34Eb–2*^ failed to complement a *Smg5* mutation (*Smg5* ^*e04233*^), which is located approximately 1 kb upstream from the 5′ region of the *Ance* gene. These results indicate that the lethality of *Ance* ^*34Eb–1*^ and *Ance* ^*34Eb–2*^ is likely due to a mutation(s) in *Smg5* rather than the *Ance* gene (data not shown). Taken together, these data suggest that *Ance* is dispensable for development.

### *Ance* mutations show genetic interaction with Dpp signaling

**Figure 4:**
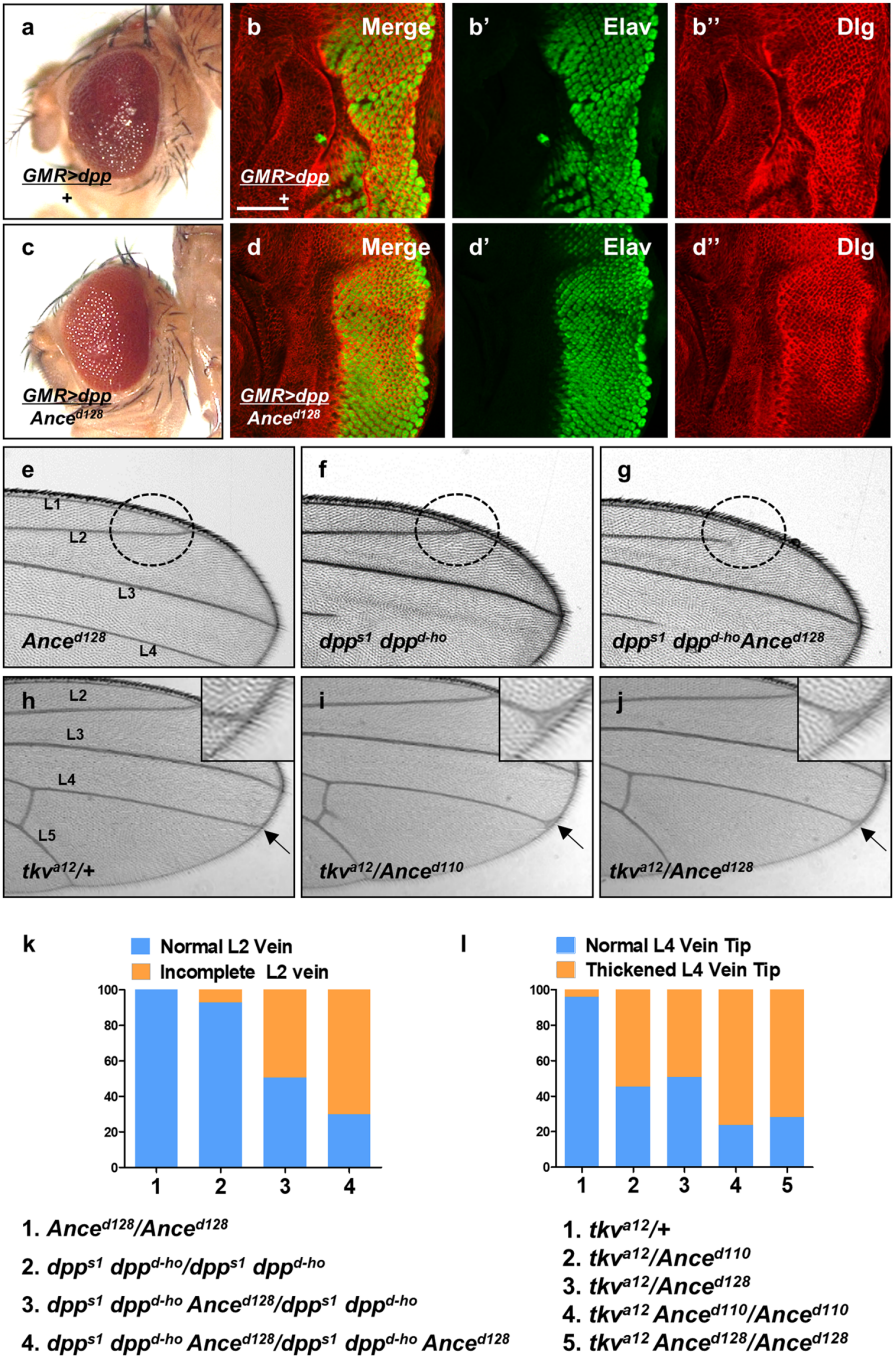
Genetic interaction between Dpp signaling and *Ance*. (**a**) Effects of *GMR* > *dpp* in adult eye. Ectopic Dpp results in roughening of the eye surface. (**b**) Effects of *GMR* > *dpp* in developing eye disc. Ectopic Dpp results in irregular wrinkling of developing retina. (b) Merge. (b’) Elav. (b”) Dlg. Scale bar, 50 μm. (**c**) Heterozygous *Ance* ^*d128*^ mutation partially suppresses *GMR* > *dpp* phenotype in adult eye. (**d**) Heterozygous *Ance* ^*d128*^ mutation partially suppresses *GMR* > *dpp* phenotype in eye disc, resulting in relatively regular arrays of photoreceptor clusters. (**e**–**g**) Genetic interaction between *dpp* and *Ance* mutations in the wing. (**e**) *Ance* ^*d128*^/*Ance* ^*d128*^ homozygote. (**f**) *dpp* ^*s1*^ *dpp* ^*d*-*ho*^/*dpp* ^*s1*^ *dpp* ^*d*-*ho*^ homozygote. (**g**) *dpp* ^*s1*^ *dpp* ^*d*-*ho*^ *Ance* ^*d128*^/*dpp* ^*s1*^ *dpp* ^*d*-*ho*^ *Ance* ^*d128*^ mutants. *dpp* ^*s1*^ *dpp* ^*d*-*ho*^ *Ance* ^*d128*^/*dpp* ^*s1*^ *dpp* ^*d*-*ho*^ *Ance* ^*d128*^ mutants show frequent partial loss of the second vein (L2 vein) as indicated by circled area. (**h**–**j**) Genetic interaction between *tkv* and *Ance* mutations. (**h**) *tkv* ^*a12*^/+ heterozygote. (*i*) *tkv* ^*a12*^/*Ance* ^*d110*^ transheterozygote. (**j**) *tkv* ^*a12*^/*Ance* ^*d128*^ transheterozygote. *tkv* ^*a12*^/*Ance* ^*d110*^ and *tkv* ^*a12*^/*Ance* ^*d128*^ transheterozygotes result in abnormal thickvein phenotype at the distal tip of the L4 vein indicated by arrow and inset. (**k**) Quantification of L2 wing vein defects in *dpp* and *Ance* mutations. Percent indicates the frequency of partial loss of L2 vein. 1 = 0% (n = 0/105), 2 = 7.3% (n = 7/95), 3 = 49.5% (n = 53/103), 4 = 70.1% (n = 68/97). (*l*) Quantification of L4 vein defects in *tkv* and *Ance* mutations. Percent indicates the frequency of the L4 vein defect. 1 = 4.1% (n = 3/71), 2 = 54.7% (n = 87/159), 3 = 49.2% (n = 67/132), 4 = 76.3% (n = 103/135), 5 = 71.8% (n = 102/142).

Although *Ance* deletion mutants and *Ance* RNAi flies show no discernible phenotype in the normal condition, it is possible that *Ance* mutations might show synthetic phenotypes with other mutations in the functionally related genes. Since *Ance* expression is regulated by Dpp signaling, we reasoned that *Ance* might show genetic interaction with Dpp signaling components. To detect genetic interaction, we examined the effects of altering the *Ance* level on the phenotypes of *dpp* overexpression or *dpp* mutations. First, we used flies that overexpress *dpp* in the eye. As shown in Fig. 4a, overexpression of *dpp* under *GMR*-*Gal4* driver (*GMR* > *dpp*) resulted in roughening of the eye surface. Under this background, reducing the *Ance* gene dosage by one copy of *Ance* mutation partially suppressed the rough eye phenotype, showing improved arrays of ommatidia (Fig. 4c). In third instar eye disc, *dpp* overexpression by *GMR*-*Gal4* resulted in irregular wrinkling of developing retina, as shown by immunostaining for the pan-neuronal marker Elav and the cell membrane marker Dlg (Fig. 4b-b”). One copy of *Ance* mutation significantly suppressed the abnormalities caused by *dpp* overexpression in eye disc (Fig. 4d-d”).

We also checked whether *Ance* mutations genetically interact with *dpp* hypomorphic mutation. *Ance* ^*d128*^ mutation does not affect any vein (Fig. 4e). *dpp* ^*s1*^ *dpp* ^*d*-*ho*^ homozygotes show loss of distal part of 4^th^ longitudinal wing vein (L4) (Fig. 4f), besides the partial loss of the L2 vein at a low frequency (7.3%) as seen in Fig. 4g. Interestingly, *Ance* ^*d128*^ mutation strongly enhanced the frequency of L2 vein loss phenotypes of *dpp* ^*s1*^ *dpp* ^*d*-*ho*^ to 70.1% of flies examined (Fig. 4g,k). This result indicates that there is a synergistic interaction between *Ance* and *dpp* mutations. In addition, we tested whether mutations in *Ance* and *tkv* ^*a12*^ can show genetic interaction in the wing. *tkv* ^*a12*^/+ heterozygote showed normal veins (Fig. 4h). However, *tkv* ^*a12*^/*Ance* ^*d110*^ trans-heterozygote resulted in a weak thickvein phenotype at the distal tip of the L4 vein (Fig. 4i, 54.7%). *tkv* ^*a12*^/*Ance* ^*d128*^ trans-heterozygote showed nearly identical genetic interaction as *tkv* ^*a12*^/*Ance* ^*d110*^ (Fig. 4j, 49.2%). In addition, *tkv* ^*a12*^ heterozygotes under *Ance* ^*d110*^ (or *Ance* ^*d128*^) homozygote condition showed increased frequencies of the thickvein phenotype (Fig. 4i, 76.3% and 71.8%, respectively). Taken together, these data show that *dpp* mutant phenotypes can be modified by altering the level of Ance function, although loss of *Ance* alone is insufficient to cause visible developmental defects.

### *Ance* expression is directly controlled by *pannier*

**Figure 5:**
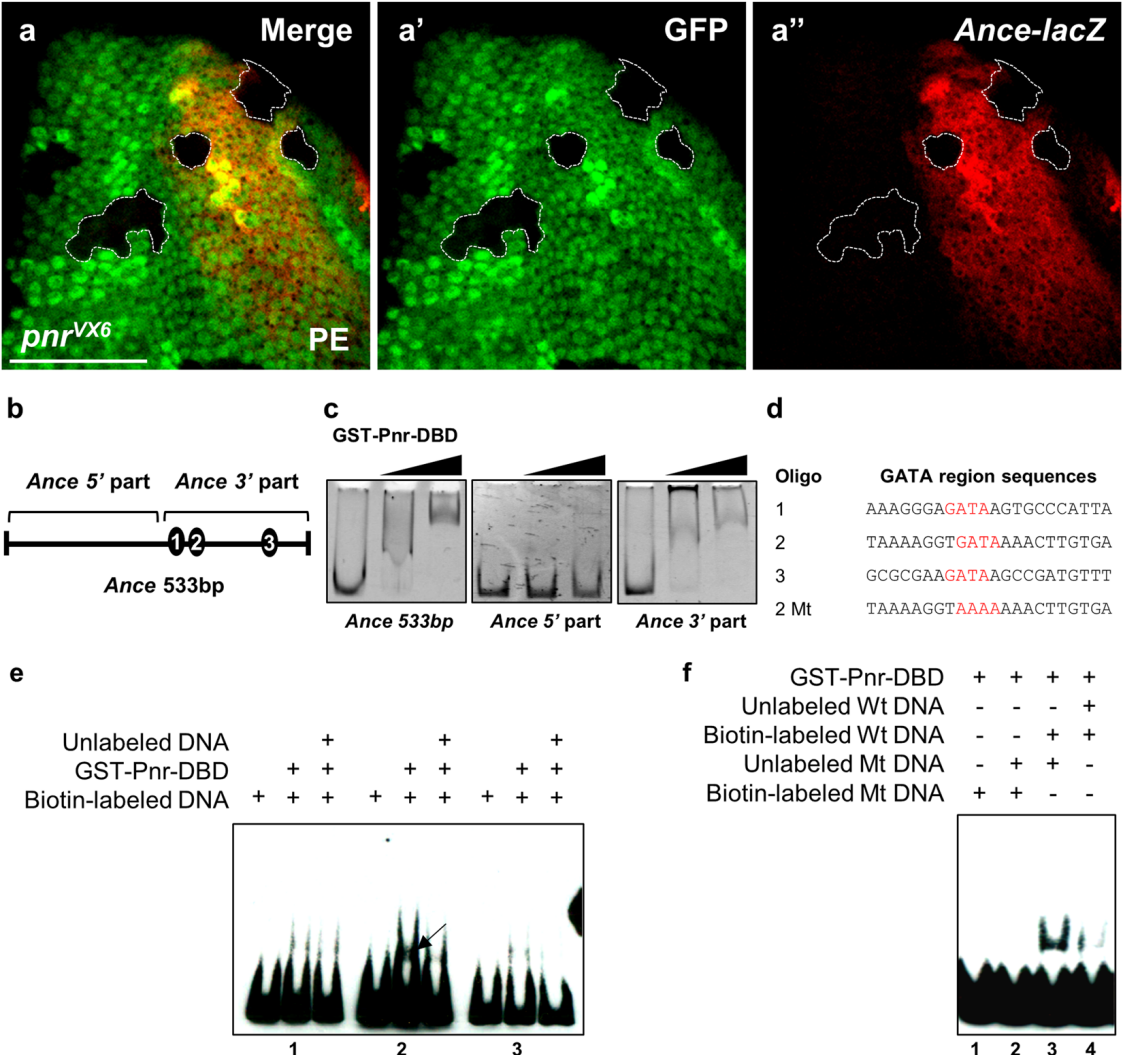
Pnr is required for *Ance* expression and binds to *Ance* regulatory region. (**a**) *pnr* ^*VX6*^ mutant clones result in reduction of *Ance*-*lacZ* expression in the dorsal eye disc. (a) Merge. (a’) GFP. (a”) *Ance*-*lacZ*. The areas marked by dotted lines show clear decrease in the level of *Ance*-*lacZ*. PE: peripodial membrane. Scale bars, 50 μm. (**b**) The scheme of a 533 bp regulatory region of *Ance*-*lacZ* reporter. This regulatory region of *Ance*-*lacZ* has three potential GATA sites, as shown in the GATA region sequences in (d). (**c**) Pnr binds to *Ance* regulatory region. Electrophoretic mobility shift assay (EMSA) shows that *Ance* 533 bp enhancer is bound by increasing amounts of GST-Pnr-DBD. Only 3′ part of *Ance* enhancer is bound by GST-Pnr-DBD. (**d**) Sequences of GATA regions in *Ance* enhancer. In *Ance 3*′ regulatory part, three potential GATA sites exist. Three oligonucleotides (oligo 1 to 3) and a mutated oligo were used in (e,f). ‘2 Mt’ indicates an oligonucleotides mutated in the GATA 2 region. (**e**) Pnr binds to a second GATA site in the *Ance* regulatory region. EMSA with biotin-labeled potential GATA sites and GST-Pnr-DBD shows considerable band upshift by the second GATA site. This band shift is inhibited by unlabeled competitor DNA. (**f**) Mutation of the second GATA site in the *Ance* regulatory region (biotin-labeled Mt DNA) results in no binding with Pnr-DBD (lane 1). Addition of the oligo mutated in the second GATA (unlabeled Mt DNA) in the lane 1 reaction has no effect (lane 2). Pnr-DBD could bind to biotin-labeled wild-type (Wt) DNA in the presence of unlabeled Mt DNA (lane 3). Unlabeled Wt oligo reduced the binding between Pnr-DB and biotin-labeled Wt DNA (lane 4).

As described earlier (Fig. 1), the *Ance*-*lacZ* reporter shows preferential dorsal expression similar to the pattern of *pannier* (*pnr*) expression^27,34,35^. To check whether *pnr* is involved in regulating *Ance* expression during larval development, we generated clones of *pnr* null mutant cells in eye imaginal discs. Because *Ance*-*lacZ* is expressed near the dorsal margin of the eye disc, *pnr* ^*VX6*^ clones outside the *Ance*-*lacZ* expressing region did not show any *lacZ* expression. Conversely, *pnr* ^*VX6*^ mutant clones within the *Ance*-expressing dorsal area of the eye disc showed little or no expression of *Ance*-*lacZ* (Figs 5a-a” and S6). In contrast, variable levels of Ance protein were detected in *pnr* ^*VX6*^ mutant clones (Fig. S7), consistent with non-cell autonomous distribution of Ance protein. We also tested the effects of Pnr overexpression using various Gal4 drivers but could not check the effects due to early lethality. Nevertheless, our analysis of mutant clones clearly indicates that *pnr* is required for the dorsal-specific *Ance* expression.

As *Ance*-*lacZ* expression was lost in *pnr* ^*VX6*^ mutant clones, we hypothesized that Pnr might be directly required for *Ance* expression. To check this possibility, we tested whether Pnr can bind to the *Ance* enhancer. The *Ance*-*lacZ* reporter contains a 533 bp *Ance* regulatory region which has three potential GATA binding sites in the 3′ half. First, we carried out electrophoretic mobility shift assay (EMSA) using purified GST-Pnr-DBD (DNA binding domain) and the 533 bp *Ance* regulatory region. EMSA using GST-Pnr-DBD and the 533 bp *Ance* enhancer showed an upward band shift when GST-Pnr was added, indicating that Pnr binds to the *Ance* region (Fig. 5c). The 3′ region of the *Ance* enhancer containing three GATA sequences showed similar band shifting, but the 5′ region did not, suggesting that one or more of the GATA sites might be involved in binding with GST-Pnr-DBD (Fig. 5c). Additional EMSA assays with three DNA fragments containing different GATA sites showed that the second GATA site was strongly bound to GST-Pnr-DBD (Fig. 5e). These data suggest that Pnr directly regulates *Ance* expression at transcriptional level by binding to the second GATA site of the *Ance* enhancer. To confirm that the second GATA site in *Ance* enhancer is important, we generated a mutant form of GATA2 fragment, which has AAAA instead of GATA in the second GATA site. In EMSA assays, Pnr binding to the GATA2 DNA was strongly reduced by the addition of unlabeled wild-type GATA2 DNA but not by the mutated GATA2 DNA (Fig. 5f). This further supports the specific binding of Pnr to the GATA2-containing DNA.

**Figure 6:**
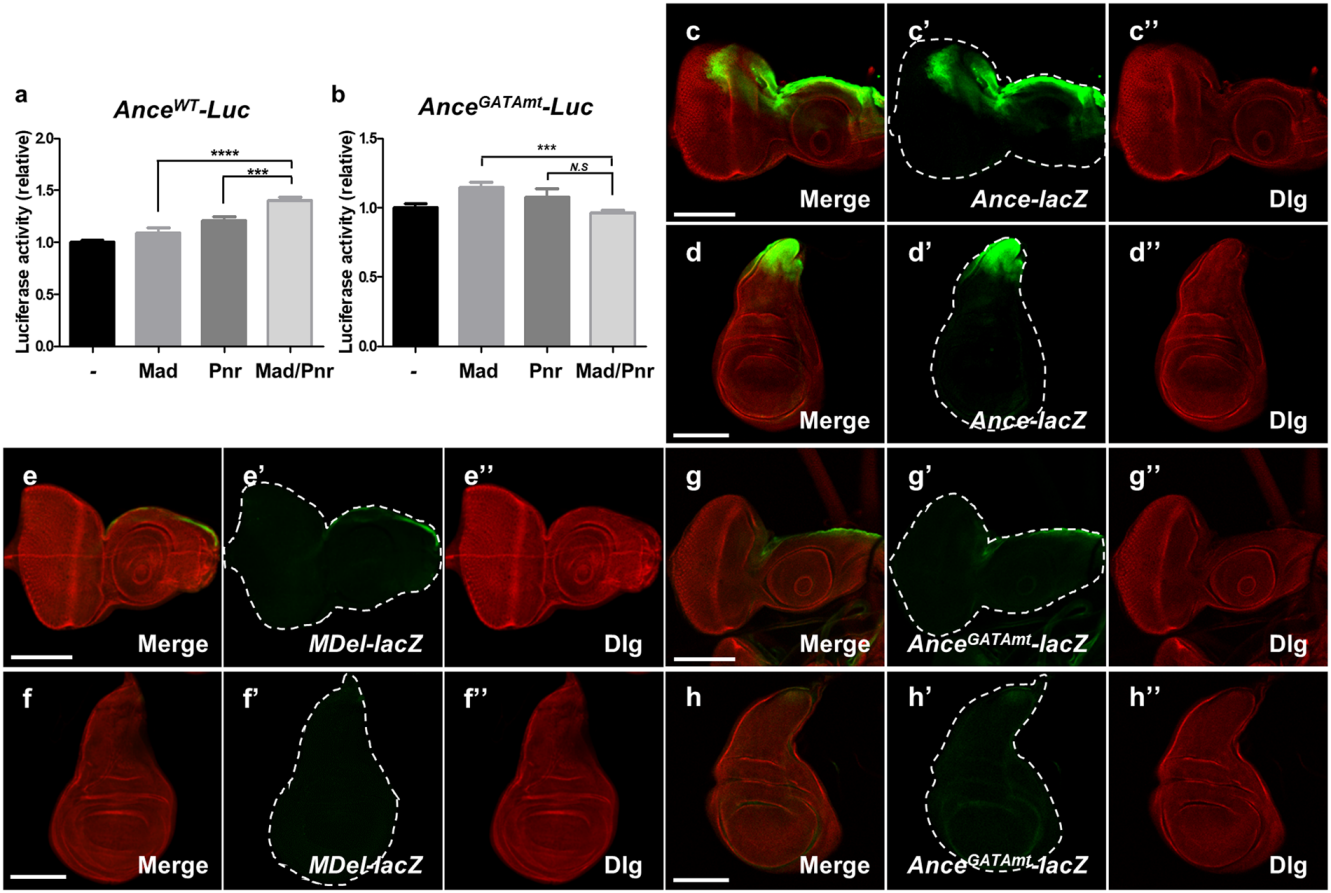
*Ance* expression is controlled by Mad and Pnr *in vivo*. (**a**) Luciferase assay using *Ance* ^*WT*^-*luc* in S2 cells. Double transfection of *Mad* and *pnr* induces stronger enhancement in luciferase activity compared to single transfection of *Mad* or *pnr*. (**b**) Luciferase assay using *Ance* ^*GATAmt*^-*luc* in S2 cells. Double transfection of *Mad* and *pnr* fails to induce luciferase activity compared to single transfection of *Mad* or *pnr*. Luciferase activity was normalized to control *Renilla* luciferase activity. All data represent the mean ± s.e.m. (error bars), and *P*-values were calculated by using Student’s *t*-test. n.s, not significant. ***P < 0.001. ****P < 0.0001. (**c**-c”) Expression pattern of *Ance*-*lacZ* in eye disc. (c) Merge of (c’) and (c”). (c’) *Ance*-*lacZ*. (c”) Dlg staining. (**d**-d”) Expression pattern of *Ance*-*lacZ* in wing disc. (d) Merge of (d’) and (d”). (d’) *Ance*-*lacZ*. (d”) Dlg staining. (**e**-e”) Expression pattern of *MDel*-*lacZ* in eye disc. Loss of Mad binding sites in *Ance* enhancer nearly abolishes *lacZ* expression in eye disc (e’). (e) Merge of (e’) and (e”). (e’) *MDel*-*lacZ*. (e”) Dlg staining. (**f**) Expression pattern of *MDel*-*lacZ* in wing disc. Loss of Mad binding sites in *Ance* enhancer nearly abolishes *lacZ* expression. (f) Merge. (f’) *MDel*-*lacZ*. (f”) Dlg. (**g**-g”) Expression pattern of *Ance* ^*GATAmt*^-*lacZ* in eye disc. Mutation of the second GATA site in *Ance* enhancer results in strong reduction of *lacZ* expression. (g) Merge. (g’) *Ance* ^*GATAmt*^-*lacZ*. (g”) Dlg. (**h**) Expression pattern of *Ance* ^*GATAmt*^-*lacZ* in wing disc. Mutation of the second GATA site in *Ance* enhancer results in strong reduction of *lacZ* expression. (h) Merge. (h’) *Ance* ^*GATAmt*^-*lacZ*. (h”) Dlg. Scale bars, 100 μm (c–h”).

In addition, we carried out luciferase assay in S2 cells using the wild-type and the mutated *Ance* GATA enhancer. Single transfection of either *Mad* or *pnr* weakly increased luciferase activity by 8.8% and 20.6%, respectively. Double transfection of *Mad* and *pnr* significantly enhanced luciferase activity to 40.3%, which was higher than the combined effects of *Mad* and *pnr* (Fig. 6a). Next, we performed similar luciferase assays with *Ance* ^*GATAmt*^, the *Ance* regulatory region with a mutation in the GATA2 site. In this assay, Mad alone could weakly activate luciferase activity (11.4%), but Pnr alone did not significantly enhance the luciferase activity due to the GATA2 mutation. Interestingly, when both *Mad* and *pnr* were overexpressed, the effect of Mad was consistently inhibited (3.8% lower than the control level) (Fig. 6b). This suggests that overexpressed Pnr may interact with Mad but fail to bind to the mutated *Ance* ^*GATAmt*^ regulatory region, perhaps dominantly inhibiting the Mad effects.

Our data from the luciferase assays in S2 cells showed that Mad and Pnr can bind to the *Ance* regulatory region to activate transcription. However, the level of activation was relatively weak, and it was difficult to determine whether the combined effects of Mad and Pnr were additive or synergistic. Thus, we tested the effects of Mad and Pnr *in vivo* using transgenic reporter lines. Firstly, to determine the effects of Dpp signaling on the *Ance* expression, we utilized an *MDel*-*lacZ* reporter, which is a modified version of *Ance*-*lacZ* mutated in the Mad-binding sites^19^. As shown in Fig. 6e and f, *MDel*-*lacZ* expression was significantly reduced in both eye and wing imaginal discs compared to *Ance*-*lacZ* (Fig. 6c,d). In addition, we checked the possibility that *Ance* regulated by Dpp signaling might have a positive or negative feedback during development. As indicated in Fig. S8, there was no difference in the level of phospho-Mad (p-Mad) between wild-type and *Ance*-deletion mutant, indicating that there is no obvious feedback regulation between Dpp signaling and Ance.

Secondly, to test whether the second GATA site in the *Ance* enhancer is important *in vivo*, we generated an *Ance* ^*GATAmt*^-*lacZ* line, which consists of AAAA instead of GATA in the second GATA site and checked its expression pattern in eye and wing discs. As indicated in Fig. 6g and h, *lacZ* expression was significantly reduced compared to *Ance*-*lacZ* expression (Fig. 6c,d), indicating that the second GATA site bound by Pnr is required for *Ance* expression. Since loss of either Mad or Pnr binding site severely reduces *Ance* expression in imaginal discs, these data suggest that Mad and Pnr act together *in vivo* rather than acting independently.

### Physical and genetic interaction between Pnr and Dpp signaling

**Figure 7:**
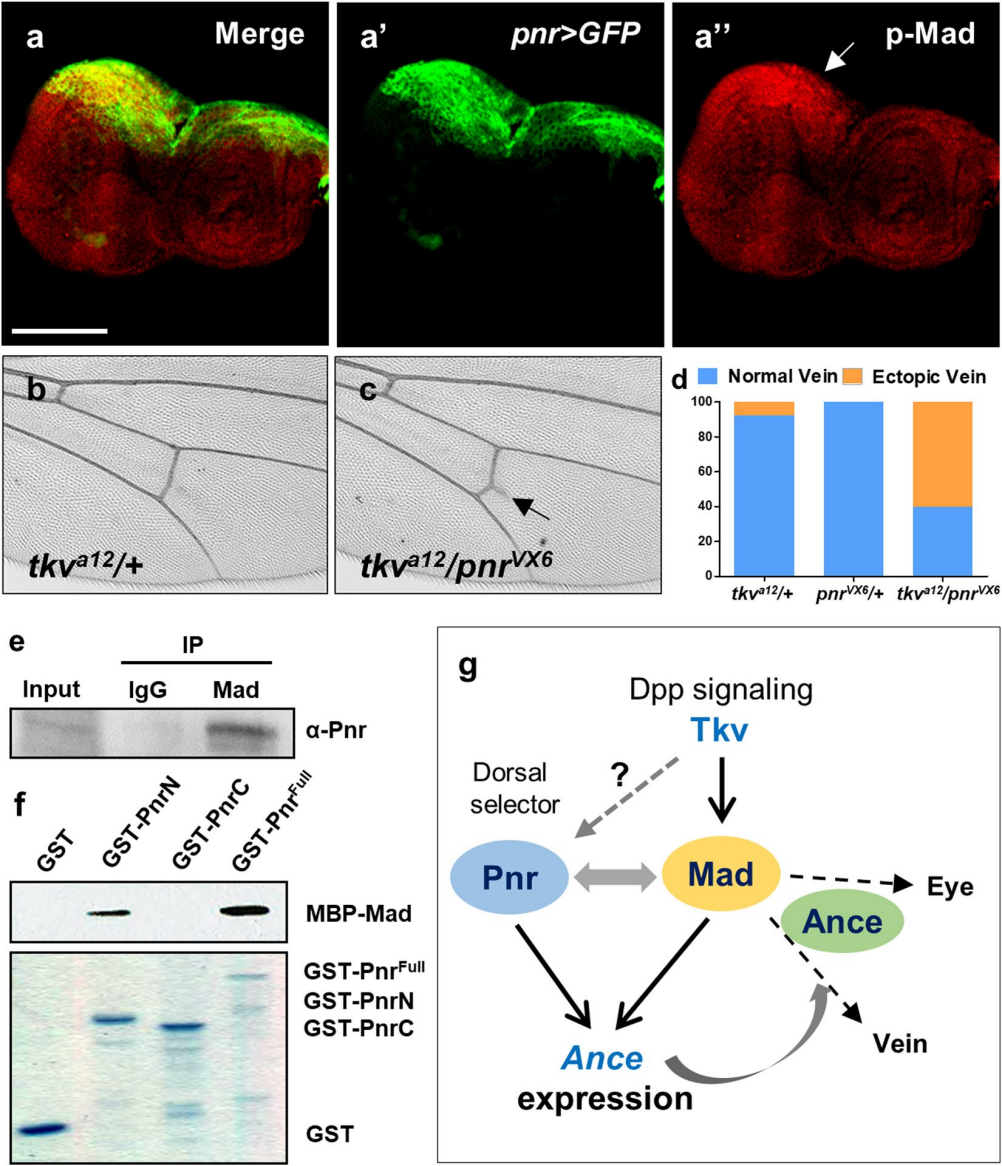
Physical and genetic interaction between Pnr and Dpp signaling. (**a**) Immunostaining of *pnr* > *GFP* eye disc for GFP and phospho-Mad (p-Mad). GFP is selectively expressed in the dorsal region. p-Mad expression is ubiquitously distributed but is relatively stronger in the dorsal region. (a) Merge. (a’) *pnr* > *GFP*. (a”) pMad. Scale bars, 100 μm. (**b-d**) Genetic interaction between *tkv* ^*a12*^ and *pnr* ^*VX6*^. *tkv* ^*a12*^/+ heterozygote shows normal posterior crossvein (c). *tkv* ^*a12*^/*pnr* ^*VX6*^ transheterozygote results in extra vein formation (indicated by arrow) from the posterior crossvein (c). (**d**) Quantification of the extra vein phenotype. Percentage of the extra vein phenotype in *tkv* ^*a12*^/+, *pnr* ^*VX6*^/+, and *tkv* ^*a12*^/*pnr* ^*VX6*^ flies. *tkv* ^*a12*^/+ = 7.9% (n = 6/76), *pnr* ^*VX6*^/+ = 0% (n = 0/75), *tkv* ^*a12*^/*pnr* ^*VX6*^ = 60.4% (n = 58/96). (**e**) Mad and Pnr form a complex. Pnr is co-immunoprecipitated by anti-Mad antibody in larval extracts. (**f**) Mad and Pnr directly interact. GST-pulldown assay shows direct interaction between Pnr and Mad. N-terminal part of Pnr physically interacts with MBP-Mad. Coomassie blue-stained SDS-PAGE gel shows GST, GST-PnrN, GST-PnrC, and GST-Pnr^Full^. (**g**) A model for the relationships among Ance, Dpp signaling, and Pnr in imaginal discs. Dorsal expression of *Ance* is regulated by a combination of three ways. Firstly, Mad activated by Dpp signaling binds to a Mad binding site of the *Ance* regulatory region. Secondly, Pnr directly activates *Ance* expression by binding to a GATA site of *Ance* regulatory region. Thirdly, Mad physically interacts with dorsal-specific Pnr, which may provide the dorsal specificity of *Ance* expression. Mad is required for wing vein and eye differentiation by activating unknown targets (dashed arrows). Genetic interactions with Mad suggest that Ance may affect the Mad function in eye and wing vein development (see Discussion for details). It is unknown whether *pnr* is regulated by Dpp signaling as in embryogenesis.

Our data described above show that Mad and Pnr might function together for *Ance* expression in imaginal discs. While *pnr* expression is restricted to the dorsal domain^27,34,35^, *dpp* mRNA expression in third instar eye disc is restricted to the morphogenetic furrow and part of the dorsal/ventral margins^21,39^. However, Dpp is secreted to broader regions of the eye disc. Therefore, although mRNA expression patterns of *pnr* and *dpp* are not identical in the eye disc, cells that receive the Dpp signal might overlap in the dorsal margin region of *pnr* expression. Thus, we examined the pattern of p-Mad expression in third instar eye disc. p-Mad expression was enriched along the MF, but it was also elevated at the dorsal margin of eye disc where *pnr* is expressed (Fig. 7a-a”, arrow). Therefore, Pnr and Mad may function together to regulate *Ance* expression. To address this possibility, we tested whether Pnr and Dpp signaling genetically interact (Fig. 7b). When *tkv* ^*a12*^ and *pnr* ^*VX6*^ flies were crossed, trans-heterozygote progeny (*tkv*/+; *pnr*/+) showed extra vein formation from the posterior crossvein (Fig. 7b,c). Although the extra vein phenotype was mild, it was consistently observed in 60.4% transheterozygotes, while such phenotype was rarely detected in *tkv*/+ or *pnr*/+ heterozygotes (7.9% and 0%, respectively) (Fig. 7d).

The overlapping localization of Pnr and Mad and their genetic interaction suggested that they might be associated together in a protein complex. To test this possibility, we carried out co-immunoprecipitation between Pnr and Mad using larval extracts. As shown in Fig. 7e, Pnr was co-immunoprecipitated by anti-Mad antibody, suggesting that Pnr and Mad physically interact. We also checked whether their interaction is direct. GST-pulldown assay using bacterially purified GST-Pnr and MBP-Mad showed that N-terminal part of Pnr binds to MBP-Mad (Fig. 7f), indicating that Pnr and Mad directly interact. Taken together, these data support physical and functional interaction between Pnr and Mad, as outlined in the scheme shown in Fig. 7g (See Discussion).

### *SMAD2* and *GATA4* are required for *ACE* regulation in human cells

**Figure 8:**
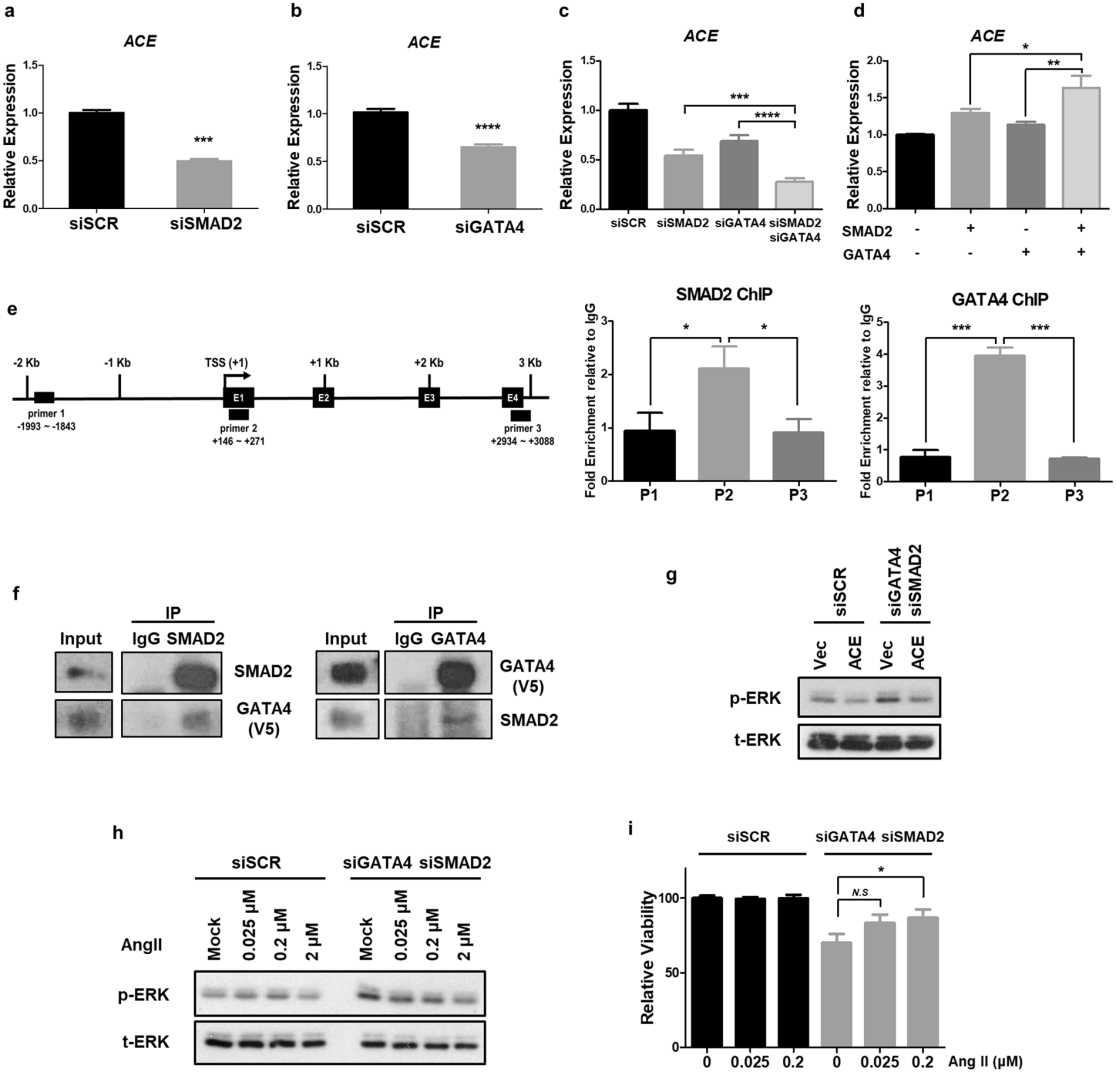
SMAD2 and GATA4 are required for *ACE* expression in HEK293 cells. (**a**–**c**) Effects of silencing *SMAD2* or *GATA4* on *ACE* expression. Y-axis indicates relative expression compared with control siRNA (*siSCR*). (**a**) *SMAD2* knockdown results in reduction of *ACE* level (49.4%). (**b**) *GATA4* knockdown reduces *ACE* level (64.9%). (**c**) Single knockdown of *SMAD2* or *GATA4* decreases *ACE* expression (54.1% or 68.9%), respectively. Double siRNAs for *SMAD2* and *GATA4* result in further inhibition of *ACE* level (26.5%). (**d**) *ACE* expression upon overexpressing *SMAD2* and/or *GATA4*. Double transfection of *SMAD2* and *GATA4*-expressing vectors induces stronger enhancement in *ACE* expression compared to single transfection of *SMAD2* or *GATA4*. (**e**) Enrichment of SMAD2 and GATA4 on *ACE* promoter. Left: Schematic diagram of human *ACE* promoter. Transcription start site (TSS) and primer sets used for qPCR are indicated. Right: Relative enrichment of SMAD2 and GATA4 to IgG control. (**f**) Reciprocal co-immunoprecipitation between SMAD2 and GATA4 in HEK293 cells. Overexpressed GATA4 (GATA4-V5) is co-immunoprecipitated with endogenous SMAD2 and *vice versa*. GATA4 is detected by anti-V5 antibody. (**g**) *ACE* overexpression suppresses the increased level of phospho-ERK (p-ERK) in *SMAD2*/*GATA4*-knockdown HEK293 cells. *siSMAD2*/*siGATA4* in HEK293 cells increases p-ERK compared to *siSCR* control. *ACE* overexpression decreases p-ERK in *siSMAD2*/*siGATA4*-treated cells. (**h**) Angiotensin II (Ang II) treatment reduces the level of p-ERK in *SMAD2*/*GATA4*-knockdown HEK293 cells. *siSMAD2*/*siGATA4* in HEK293 cells increases p-ERK compared to *siSCR* control. Ang II treatment decreases p-ERK in a dose-dependent manner. (**i**) Ang II rescues the reduced cell viability in *SMAD2*/*GATA4*-knockdown HEK293 cells in a dose-dependent manner. All data represent the mean ± s.e.m. (error bars) and *P*-values were calculated by using Student’s *t*-test. n.s, not significant, *P < 0.05. **P < 0.01. ***P < 0.001. ****P < 0.0001.

Mammalian Angiotensin-converting enzyme (ACE) plays key roles in regulating renin-angiotensin system. Since our data showed that *Drosophila* Mad and Pnr are required for *Ance* expression, we hypothesized that mammalian SMAD and GATA factors, homologs of *Drosophila* Mad and Pnr, respectively, might be involved in *ACE* expression. To test this possibility, we examined the effects of RNAi of *SMAD1*/*2* and *GATA2*/*4* in HEK293 cells (human embryonic kidney 293 cells). *SMAD1* and *GATA2* siRNAs did not affect *ACE* expression (Fig. S9a), but *SMAD2* and *GATA4* siRNAs were effective in reducing *ACE* expression (Fig. 8a,b). The levels of knockdown for each siRNA for *SMAD1*/*2* and *GATA2*/*4* were approximately 70–90% (Fig. S9b,c). Furthermore, double siRNA treatments for both *SMAD2* and *GATA4* were more effective in reducing *ACE* expression compared to single siRNA for *SMAD2* or *GATA4* (Fig. 8c). We also tested whether overexpressing *SMAD2* and *GATA4* is sufficient to induce the expression of *ACE*. Overexpression of either *SMAD2* or *GATA4* showed slight increase in the *ACE* mRNA level, whereas overexpression of both *SMAD2* and *GATA4* resulted in further increase (Fig. 8d), which was similar to the effects of overexpressing Mad and Pnr in *Drosophila* S2 cells (Fig. 6a).

To check whether SMAD2 and GATA4 are directly involved in regulation of *ACE* expression, we performed chromatin immunoprecipitation (ChIP) assays (Fig. 8e). Our data showed that GATA4 and SMAD2 bind to the first exon of *ACE* gene, indicating their transcriptional roles in the regulation of *ACE* expression. Taken together, both SMAD2 and GATA4 are required for *ACE* expression in HEK293 cells as in *Drosophila*.

The regulation of *ACE* expression by SMAD2 and GATA4 suggests that SMAD2 and GATA4 might be associated in a protein complex to regulate their target gene expression as in *Drosophila*. To test this possibility, we carried out co-immunoprecipitation experiments between SMAD2 and GATA4 using GATA4-overexpressing HEK293 cell lysates. Overexpressed GATA4 was co-immunoprecipitated by endogenous SMAD2 immunoprecipitation, and endogenous SMAD2 was also co-immunoprecipitated by overexpressed GATA4 (Fig. 8f). These results suggest that SMAD2 and GATA4 form a protein complex in human as seen in the Mad-Pnr co-immunoprecipitation in fly.

To look for clues to the function of SMAD2 and GATA4, we examined their effects on the extracellular signal-regulated kinase (ERK) signaling that regulates a variety of cell physiology. First, we checked the level of phosphorylation of ERK (p-ERK) and found that the treatment of *siSMAD2*/*siGATA4* increased p-ERK compared to the *siScramble* (*siSCR*) control (Fig. 8g). Interestingly, the increased p-ERK level in *SMAD2*/*GATA4*-knockdown HEK293 cells was suppressed when their target gene *ACE* was overexpressed. We also tested whether an addition of Angiotensin II (Ang II) can have similar effects. Indeed, the increased p-ERK level in *SMAD2*/*GATA4*-knockdown cells was antagonized by Ang II in a dose-dependent manner, indicating that *ACE* overexpression and Ang II treatment could rescue the effects of silencing *SMAD2* and *GATA4*. Next, we checked viability in HEK293 cells. As shown in Fig. 8i, *SMAD2* and *GATA4* siRNAs decreased cell viability compared to control siRNA. An addition of Ang II did not affect the viability in *siSCR*-treated HEK293 cells, but it partially rescued reduced cell viability of *SMAD2*/*GATA4*-knockdown cells. Taken together, we conclude that SMAD2 and GATA4 directly regulates *ACE* expression as in *Drosophila* and loss-of-function phonotypes of *SMAD2* and *GATA4* can be restored in part by *ACE* overexpression or Ang II treatment.

## Discussion

Expression of the *Drosophila* ACE homolog Ance is developmentally regulated in larval imaginal discs. Here we have provided evidence that Mad and Pnr transcription factors play key roles in *Ance* regulation.

Previously, it has been shown that the *Ance* expression during embryogenesis is activated by Dpp signaling from the ectodermal dorsal midline^10,11^. Our data show that Dpp signaling is essential for *Ance* expression in imaginal discs, consistent with the role of Dpp for *Ance* expression in embryogenesis. Transcriptional activation in eukaryotes is often regulated by synergistic effects of other enhancer binding proteins^40^. It has been shown that Mad is a partner of the Fos transcription factor for a context-dependent transcriptional activation in *Drosophila* ^41^. Mammalian Smad proteins can also promote different TGFβ target gene expression in cooperation with cell type-specific transcription factors^42,43^. In this regard, it is worth noting that both Mad and the GATA factor Pnr are required for *Ance* expression during larval development. In embryogenesis, *Ance* and *pnr* are independent targets of Dpp signaling with different thresholds for Dpp activity. In contrast, *Ance* expression in imaginal discs is regulated by a combination of Dpp and Pnr.

According to the clonal analysis, loss of either Mad or Pnr almost completely abolishes the *Ance*-*lacZ* expression in the eye and wing discs. This indicates that Mad and Pnr regulate *Ance* expression in a cooperative manner, consistent with their physical interaction. We also found a weak but consistent genetic interaction in wing venation between *tkv* and *pnr* mutations (Fig. 7b–d). Based on these results, we propose a model in which Mad activated by Dpp signaling can interact with Pnr for tissue-specific *Ance* expression (Fig. 7g). Secreted Dpp activates Mad in broad regions of imaginal discs. In contrast, *Ance* expression is enriched in the dorsal area of the eye and wing discs, indicating that additional dorsal factors are required for the restricted *Ance* expression. In our model, one of such dorsal factors might be Pnr, thus allowing context-dependent expression of the *Ance* gene in the dorsal-specific pattern.

The *Ance* deletion mutants generated in this study develop normally to fertile adults. This result was somewhat unexpected because *Ance* mutants were reported to be lethal or sterile^10,31,33^. Our molecular and genetic analysis of *Ance* null mutants shows that Ance is dispensable for development and fertility. It is possible that other Ance homologs might be able to compensate the loss of Ance. Ance and its homolog, Angiotensin-converting enzyme-related (Acer), can catalyze the same peptide, although they have different affinities for the substrates^44^. Therefore, there is a possibility that the Acer function could compensate for loss of Ance during development. However, double mutants for *Ance* and *Acer* were also found to be developmentally normal and fertile (data not shown). Analysis of the Ance function is more complicated due to the presence of four other *Ance*-related genes in the fly genome; *Ance*-*2*, *Ance*-*3*, *Ance*-*4*, and *Ance*-*5*. Due to their potentially redundant functions, loss of one or more Ance homologs may not affect animal development, unless the functions of all the Ance homologs are disrupted. Alternatively, other peptidases may also be used in the development of imaginal discs to circumvent the absence of Ance peptidase activity. Imaginal discs contain a variety of peptidases that hydrolyze a wide range of peptides and proteins^45^. The hydrolysis of peptides and proteins by some of these peptidases might be important for the production of dipeptides and free amino acids for protein synthesis required for morphogenesis. Active forms of signaling peptides might also be generated by peptidases for development of imaginal discs^44,45^. Thus, the absence of Ance can be compensated by other peptidases that can hydrolyze unidentified substrates of Ance to produce necessary peptides for development.

Although *Ance* null mutants do not exhibit obvious developmental defects, it is important to note that loss of *Ance* can modulate the phenotypes of impaired Dpp signaling. It has been shown that overexpression of *dpp* by *GMR*-*Gal4* causes severe roughness and bulging of the adult eye^46^. We showed that reduced *Ance* partially suppressed the effects of Dpp overexpression (Fig. 4a–d). These results suggest that the effects of *dpp* gain-of-function in the eye are mediated at least in part by Ance, consistent with the induction of *Ance* gene expression by Dpp signaling. Because *Ance* overexpression alone is insufficient to affect the eye, Ance seems to modulate the function of an unknown target of Dpp signaling to modify the Dpp-induced eye phenotype (Fig. 7g). Because Ance protein is detected in the eye disc lumen as well as disc proper cells, it may produce peptide factors that modify the effects of Dpp shown in Fig. 4c,d. Furthermore, our data show genetic interaction between hypomorphic *dpp* (or *tkv*) mutations and *Ance* mutation in wing veins. *Ance*-*lacZ* is enriched in the dorsal part of wing disc, but high levels of Ance protein are broadly detected in the lumen of the wing pouch area (Fig. 1d’,d”, Fig. S2k,l). Ance protein abundant in the wing pouch, either in the lumen or disc proper cells by uptake, may produce active peptides to affect vein differentiation, thus providing a possible explanation for the genetic interactions in vein phenotypes. These genetic interactions in *dpp Ance* double mutants (*dpp Ance*) are synergistic, since *dpp* or *Ance* single mutants show little deficiency in the second vein (Fig. 4e–g,k). Under *dpp* (or *tkv*) hypomorphic conditions, the *Ance* expression level is expected to be reduced partially. The introduction of an *Ance* null allele under this condition further reduces the Ance level. However, because *Ance* null mutants develop normally, the vein phenotype in *dpp Ance* double mutants cannot simply be explained by a reduction of the Ance level. Instead, Ance may modulate a Mad function in vein patterning. When Dpp signaling is fully functional, the Mad function in vein development may not be affected by loss of Ance alone. In *tkv* heterozygotes or *dpp* hypomorphic conditions, however, the reduction of Ance may critically impair such pathways as seen in the double mutant conditions. Thus, despite the lack of visible phenotypes in *Ance* null mutants, Ance seems to play modulatory roles when Dpp signaling is partially compromised. These genetic results raise a possibility that *Ance* expression is not only regulated by Mad but also interacts with specific Mad signaling outputs that affect diverse tissues, as proposed in Fig. 7g. The identification of related substrates for Ance dipeptidase activity will help to understand the mechanism underlying these genetic interactions.

The human somatic ACE, a zinc-dependent dipeptidyl carboxypeptidase, plays an important role in the regulation of the renin-angiotensin system. ACE has long been targeted for effective treatment of hypertension and related cardiovascular diseases through ACE inhibitors^47–49^. TGFβ is also associated with activity of ACE in renal fibrosis and disease progression. Thus, anti-TGFβ antibody can improve renal fibrosis in mouse model^50^. Kidney diseases can be treated more effectively when both ACE and TGFβ are blocked by inhibitor and antibody, respectively^51^, suggesting an association between ACE and TGFβ signaling. In addition, GATA4 activates atrioventricular canal-specific enhancer that has synergistic effects with Smad during heart development^52^, although the mechanism underlying the synergy is unknown. Our data show that human SMAD2 and GATA4 are not only required for *ACE* expression but can also induce it in HEK293 cells. Our ChIP data and co-immunoprecipitation data suggest that SMAD2 and GATA4 physically interact each other and bind to the first exon of *ACE* gene to play a transcriptional role in *ACE* expression.

The Ras/Raf/ERK pathway plays oncogenic roles in many tumors^53,54^. However, ERK activation can also lead to anti-proliferative events depending on the conditions of signal activity^55,56^. According to our data, downregulation of *SMAD2* and *GATA4* in HEK293 cells reduces cell viability, possibly due to increased cell death. Increased p-ERK levels in *SMAD2*/*GATA4*-knockdown cells suggest that cell viability may be reduced by ERK-dependent apoptosis. These data are consistent with recent studies in which ERK signaling induces apoptosis in HEK 293 cells^57^. Interestingly, Angiotensin II treatment on *SMAD2*/*GATA4*-knockdown cells can rescue reduced cell viability and restore the phosphorylation level of ERK. In addition, *ACE* overexpression decreased p-ERK in *SMAD2*/*GATA4*-knockdown cells. Together, our work in *Drosophila* and human cells provides insight into the possible regulation of *ACE* expression by these two transcription factors in mammals.

## Materials and Methods

### Fly genetics

All *Drosophila* strains were grown and maintained at 25 °C. *UAS*-*dpp*, *UAS*-*tkv* ^*QD*^, *UAS*-*GFP*, *GMR*-*Gal4*, and *ptc*-*Gal4* were used for GAL4-UAS system^38^. *Ance*-*lacZ* and *Mdel*-*lacZ* lines were provided by Mike Levine (Princeton University, USA) and Christine Rushlow (New York University, USA), respectively. *dpp* ^*s1*^ *dpp* ^*d*-*ho*^ (Bloomington, stock #398), *tkv* ^*a12*^ *FRT40A*/*CyO* (gift from Christian Dahmann, Technische Universität, Dresden, Germany), *Mad* ^*1-2*^ *FRT40A*/*CyO*, *hsflp; ubi*-*GFP FRT40A*, *FRT82B pnr* ^*VX6*^/*TM6B*, *Tb*, *hsflp; FRT82B ubi*-*GFP*, *hsflp; FRT82B arm*-*lacZ* lines were used for checking genetic interaction and generating mutant clones. For generation of *Ance* deletion lines (*Ance* ^*d110*^ and *Ance* ^*d128*^), *P*(*EP*)*G3223* was imprecisely excised by crossing with *Bc*/*CyO*, *Δ2*-*3* flies. For generation of *Ance* ^*GATAmt*^-*lacZ* line, *Ance* promoter with mutation in GATA site was cloned into pH-Pelican vector using one-step SLIC method^58^ and the cloned vector was used for generating transgenic lines.

### Immunostaining

Eye and wing imaginal discs were fixed with 4% paraformaldehyde in PBS for 15 min on ice. After washing twice with PBS, fixed discs were blocked in 5% normal goat serum/PBT (PBS with 0.3% Triton-X100) for 30 min. Samples were incubated at 4 °C overnight with primary antibodies at the following concentrations: rabbit anti-Ance (from R. Elwyn Isaac, University of Leeds, UK) at 1:2000, rabbit anti-Dlg (from Kyungok Cho, Korea Advanced Institute of Science and Technology, Korea) at 1:500, rat anti-Elav (Developmental Studies Hybridoma Bank, DSHB) at 1:100, mouse anti-lacZ (DSHB) at 1:100, sheep anti-GFP (Bio-Rad, 4745-1051) at 1:100, rabbit phospho-Smad (from Carl-Henrik Heldin, Uppsala University, Sweden) at 1:1000. After washing three times with PBT, secondary antibodies conjugated with Cy3 (1:600), Cy5 (1:400), or FITC (1:100) (Alexa Fluor, Molecular Probes) were used. Confocal fluorescent images were acquired using a Carl Zeiss LSM710 confocal microscope.

### Electrophoretic Mobility Shift Assays (EMSA)

EMSA was carried out with GST-Pnr-DBD, PCR products of *Ance* 533 bp enhancer, 5′ *Ance* enhancer, 3′ *Ance* enhancer, and biotin-labeled oligonucleotides with potential GATA binding sites. DNA binding domain of *pnr* cDNA was PCR-amplified and cloned into pGEX-4T1 vector using one-step SLIC method^58^. After transformation of pGEX-4T1-Pnr-DBD into BL21 competent cells, GST-Pnr-DBD protein was purified by using GST bead. 533 bp full-length enhancer, 5′ enhancer, and 3′ enhancer of *Ance*-*lacZ* enhancer were amplified by PCR reaction. 1 ng of *Ance* enhancer PCR products was incubated with 0.5 or 1 μg of GST-Pnr-DBD proteins in binding buffer (24 mM HEPES pH 7.9, 8 mM Tris pH 8, 2 mM EDTA, 12% glycerol, and 50 ng/μl Salmon sperm DNA) for 30 min at room temperature. After incubation, DNA-protein complexes were resolved on a 6% native PAGE gel for 1 h at 70 V in 0.5X TBE. DNA-protein complexes on native gel were visualized by RedSafe.

For EMSA with biotin-labeled oligonucleotides and GST-Pnr-DBD, biotin-labeled probes with potential GATA sites were synthesized from Bioneer (Korea). EMSA was carried out according to the method described above. After electrophoresis, DNA-protein complexes were transferred to nylon membrane. Further steps were proceeded using LightShift Chemiluminescent EMSA kit (Thermo Scientific) according to the manufacturer’s instruction.

### Luciferase assay

533 bp of wild-type or mutant *Ance* regulatory region from *Ance*-*lacZ* enhancer was cloned into pGL4.20-luc2 vector, and pAc5.1-FLAG-Mad and pAc5.1-Pnr-V5/His were generated by using pAc5.1-V5/HisA vector, *Mad* cDNA and *pnr* cDNA. Luciferase assay was performed in S2 cells using 0.1 μg pGL4.20-Ance-luc2 (or pGL4.20-Ance^GATAmt^-luc2), 0.1 μg pGL4.82-hRluc, 0.4 μg pAc5.1-FLAG-Mad, 0.4 μg pAc5.1-Pnr-V5, or 0.4 μg or 0.8 μg pAc5.1-lacZ. A total of 1 μg DNA was used for each transfection. Dual Luciferase Reporter Assay System (Promega) was used according to manufacturer’s instructions. *Firefly* luciferase activity was normalized to control *Renilla* luciferase activity.

### Immunoprecipitation

*Drosophila* larval lysates were prepared in CHAPS buffer (20 mM HEPES pH 7.5, 150 mM NaCl, 1 mM EDTA, 0.1% CHAPS, and protease inhibitor cocktail), and the lysates were cleared by centrifugation. The cleared lysate was incubated with 1 μg of goat anti-Mad antibody (Santa Cruz) for 1 hour at 4 °C. The mixture of lysate and antibody was incubated with 30 μl of Protein G Dynabead (Thermo Scientific) for overnight at 4 °C. After several rounds of washing in CHAPS buffer, proteins were eluted in SDS sample buffer by boiling at 94 °C for 5 min. Mouse anti-Pnr antibody and secondary anti-mouse antibody conjugated with HRP (Jackson ImmunoResearch) were used for western blotting.

HEK293 cell lysates were prepared in lysis buffer (40 mM Tris-Cl pH7.5, 120 mM NaCl, 1 mM EDTA, 0.3% CHAPS, protease inhibitors). The cleared lysate was incubated with 2 μg of rabbit anti-SMAD2 antibody (Cell Signaling, 5339 P) or mouse anti-GATA4 antibody (Santa Cruz, sc-25310X) for overnight at 4 °C. The mixture of lysate and antibody was incubated with 30 μl of Protein A or G SureBead (Bio-Rad) for 2 hours. Mouse anti-V5 (Invitrogen, R960-25) and Rabbit anti-SMAD2 were used for western blot.

### GST pull-down assay

Full-length, N-terminal, and C-terminal parts of *pnr* cDNA were cloned into pGEX-4T1 vector using one-step SLIC method. Full-length Mad cDNA was cloned into pMal-c2 vector. For GST-pulldown, IPTG-inducible R2 cells (BL21 derivative) were transformed with plasmids for GST-Pnr^Full^, GST-Pnr^N-term^, GST-Pnr^C-term^, and MBP-Mad. GST- or MBP-proteins were purified using a standard method. Equal amount of glutathione Sepharose 4B beads (Bioprogen) with GST- and MBP-fusion proteins were incubated in pull down buffer (20 mM TrisHCl, pH 7.5, 150 mM NaCl, 0.5 mM EDTA, 10% glycerol, 1% Triton X-100, 1 mM DTT, protease inhibitor cocktail, and 1 mM PMSF). Mouse anti-MBP antibody (NEB, E8032) and secondary anti-rabbit antibody (Jackson ImmunoResearch) were used for western blotting.

### Cell culture and transfection

*Drosophila* S2 cells were cultured in M3 media (Sigma) with 10% insect medium supplement (Sigma). Transfection was carried out with Effectene reagent (Qiagen, Hilden, Germany) according to manufacturer’s instructions.

HEK293 cells were cultured in Dulbecco’s modified Eagles medium (DMEM) with 10% fetal bovine serum (FBS). For siRNA-mediated gene silencing, siRNA was transfected into HEK293 cells by reverse transfection using Lipofectamin 2000 to a final concentration of 10 μM. siRNAs were generated from Bioneer (Korea). siRNA sequences are shown in Table S1. Total RNA of siRNA-transfected cells was extracted 48 hours after transfection by Qiazol (Qiagen, UAS) according to the manufacturer’s instruction. For plasmid transfection, pCEP4 SMAD2 (Addgene, #16484) and tetO-GATA4 (Addgene, #46030) were used. cDNA was synthesized using High Capacity cDNA Reverse Transcription Kit (Applied Biosystems). Realtime PCR Master Mix (Toyobo, Japan) was used to perform qRT-PCR assays. qRT-PCR primer sequences are shown in Table S2.

*ACE* vector (Sino Biological, HG11598-UT) was used for overexpression experiment and western blot analysis, and Angiotensin II (Sigma-Aldrich, A9525) was treated for western blot analysis and cell viability assay. For western blot analysis, rabbit anti-ERK (Cell Signaling, 4595 T) and rabbit anti-phospho ERK (Cell Signaling, 3101 S) were used.

### ChIP assays

ChIP assays were performed by using a standard protocol (Millipore) with mouse anti-GATA4 (Santa Cruz, sc-25310 X), control mouse IgG, rabbit anti-SMAD2 (Cell Signaling, 5339 S), or control rabbit IgG. Eluted DNAs were analyzed by qPCR. The primer sequences are provided in Supplementary Table S3.

### Cell viability assay

Approximately 1000 cells were plated onto 96 well plates in 10% FBS media. The culture media were exchanged using 0.5% FBS media, and Ang II was treated at indicated concentration 24 hours after plating. Relative cell viability was measured 24 hours after Ang II treatment using CellTiter-Glo Luminescent Cell Viability Assay (Promega) according to the manufacturer’s instructions.

### Statistical analysis

Statistical significance was determined by unpaired one-tailed Student’s t-test. P-values of <0.05 were considered as statistically significant. All data represent the mean ± s.e.m. (error bars).

## Electronic supplementary material

Angiotensin-converting enzyme Ance is cooperatively regulated by Mad and Pannier in Drosophila imaginal discs
