# Supplementary material for: Angiotensin-converting enzyme Ance is cooperatively regulated by Mad and Pannier in Drosophila imaginal discs
Source: Sci Rep. 2017 Oct 13;7:13174. doi: 10.1038/s41598-017-13487-w (PMC5640665; doi:10.1038/s41598-017-13487-w)
Supplement: Supplementary file 1 — Angiotensin-converting enzyme Ance is cooperatively regulated by Mad and Pannier in Drosophila imaginal discs [file 41598_2017_13487_MOESM1_ESM.pdf]

Supplementary Figures and Tables

Angiotensin-converting enzyme Ance **is cooperatively regulated by Mad and Pannier in** ***Drosophila*** **imaginal discs**

Ah-Ram Kim[1], Eun-Bee Choi[1], Mi-Young Kim[1], and Kwang-Wook Choi[1,*]

[1]Department of Biological Sciences, Korea Advanced Institute of Science and Technology (KAIST), Daejeon 305-701, Republic of Korea

*Correspondence: kchoi100@kaist.ac.kr (K.W. C.)

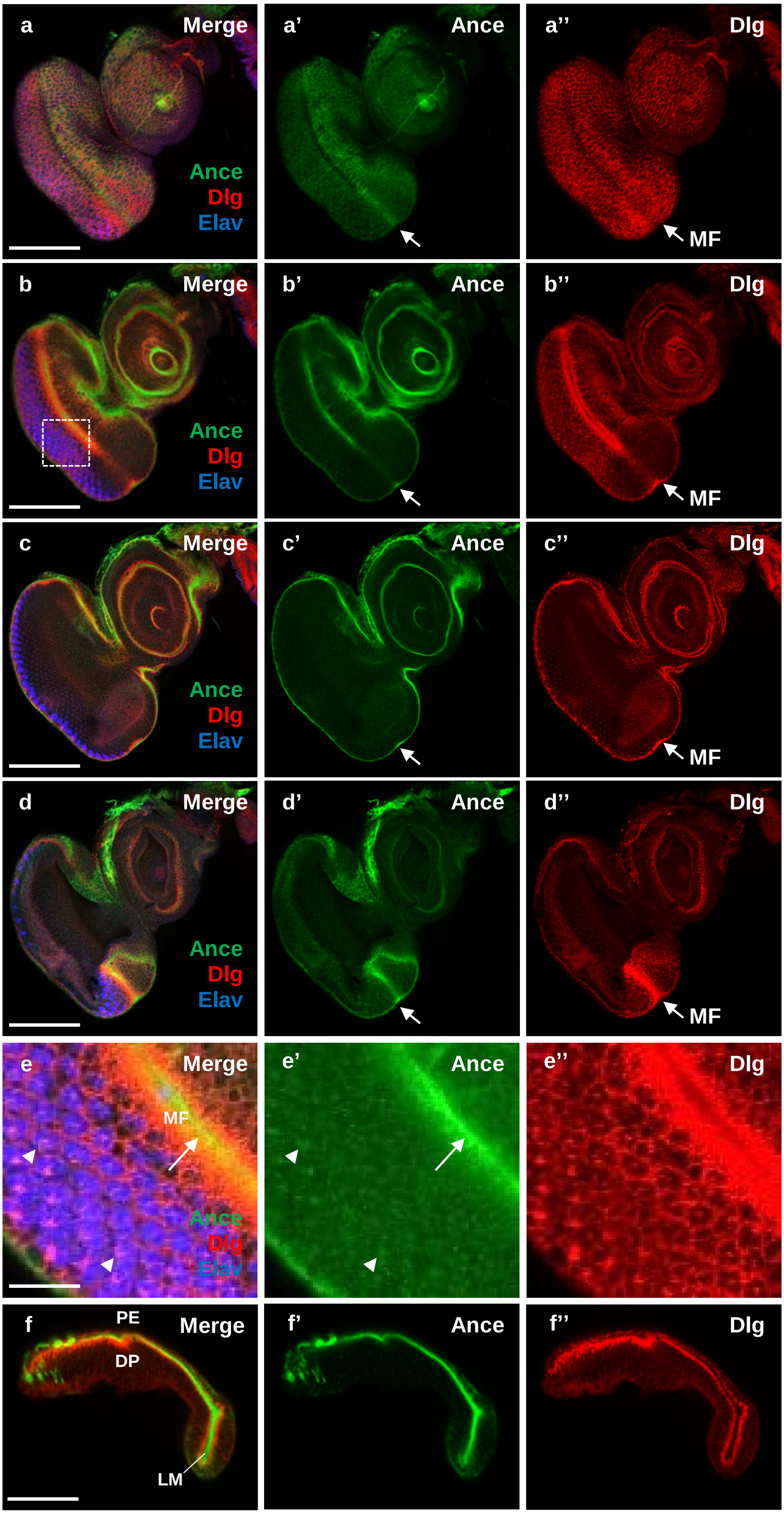

a
Merge
Ance
Dlg
Elav
a'
Ance
a''
Dlg
MF
b
Merge
Ance
Dlg
Elav
b'
Ance
b''
Dlg
MF
c
Merge
Ance
Dlg
Elav
c'
Ance
c''
Dlg
MF
d
Merge
Ance
Dlg
Elav
d'
Ance
d''
Dlg
MF
e
Merge
MF
Ance
Dlg
Elav
e'
Ance
e''
Dlg
f
PE
Merge
DP
LM
f'
Ance
f''
Dlg

Fig. S1. Expression pattern of *Ance-lacZ* and Ance protein in eye disc.

(a-d’’) Low magnification views of Ance expression in the eye-antenna disc stained by antibodies for Ance, Dlg and the neuronal marker Elav. (a) Peripodial membrane. (b) Apical disc proper. (c) Middle disc proper. (d) Basal disc proper. Ance protein is strongly detected in the lumenal space between PE and DP layers. Ance is enriched in the morphogenetic furrow (MF, arrow). (a-d) Merge. (a’-d’) Ance protein expression. (a”-d’’) Dlg staining.

(e-e”) Higher magnificent image of the boxed area in (b) shows Ance expression in the lumen along the MF. (e) Merge. (e’) Ance protein expression. Arrowheads indicate weak levels of Ance detected in the photoreceptor clusters. (e”) Dlg.

(f) Cross-section of an eye disc along the anterior posterior axis. Ance is enriched in the lumenal space between PE and DP. (f) Merge, (f’) Ance, (f’’) Dlg. PE: peripodial epithelium, DP: disc proper, LM: lumen. Scale bars, 100 μm (a-d), 20 μm (e), 50 μm (f).

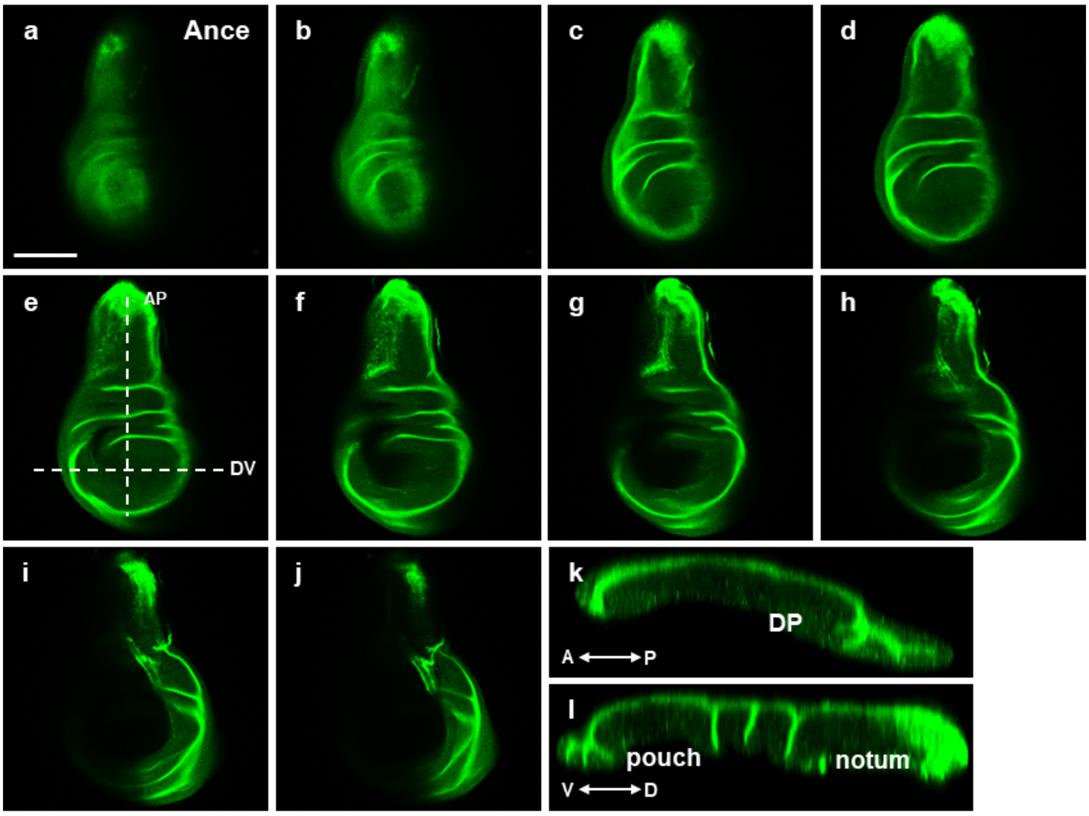

Fig. S2. Expression pattern of Ance protein in wing disc.

(a-j) Ance protein in wing disc. A series of sections from apical to basal regions show lumenal Ance proteins. Scale bar, 100 μm

(k-l) Orthogonal sections of the wing pouch region shown in (e). (k) Anterior-posterior (AP) axis. (l) Dorso-ventral (DV) axis. Note that Ance expression covers the entire DP area of wing pouch, hinge and notum in both AP and DV axis. DP: disc proper.

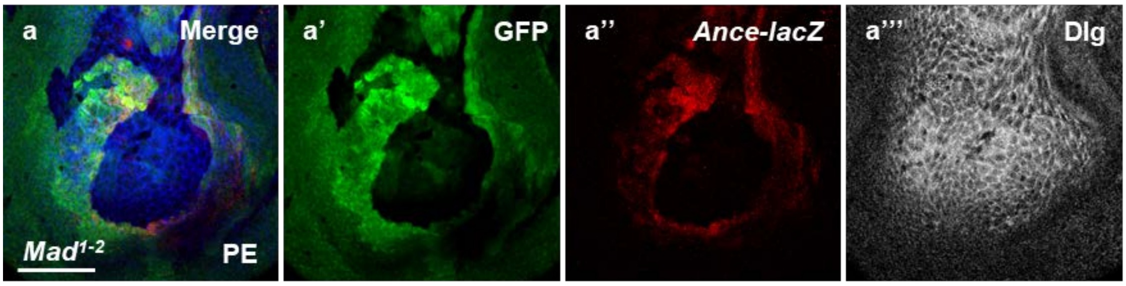

Fig. S3. Dpp signaling is required for *Ance-lacZ* expression.

(a) *Ance-lacZ* expression in *Mad*$^{1\text{-}2}$ clones is nearly absent in wing pouch. PE level section of the disc. (a) Merge, (a') GFP, (a'') *Ance-lacZ*, (a''') Dlg. Scale bar, 50 µm

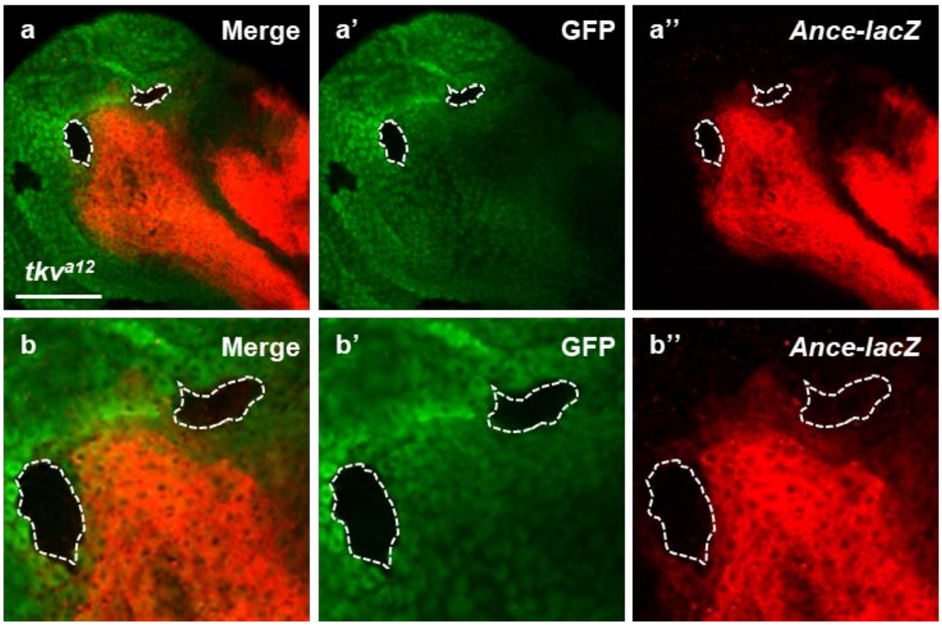

Fig. S4. Dpp signaling is required for *Ance-lacZ* expression.

(a-b) *Ance-lacZ* expression in tkv^{a12} clones is lost in peripodial epithelium of the dorsal eye disc. Mutant clones are marked by the dotted line. (a and b) Merge, (a' and b') GFP, (a'' and b'') *Ance-lacZ.*

(b) High magnification views of tkv^{a12} clones shown in (a).

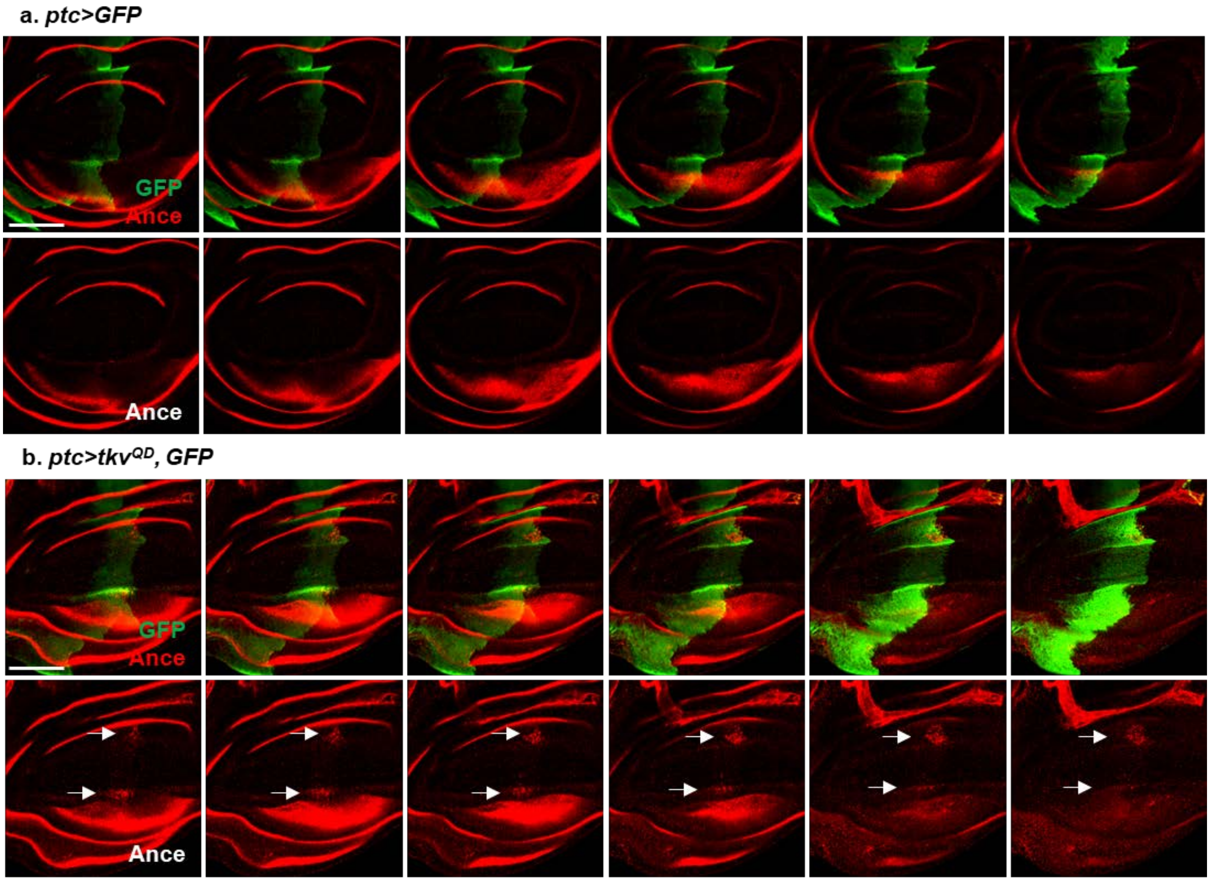

Fig. S5. Ectopic expression of *tkv*^{QD} induces Ance expression

(a) Control wing disc carrying *ptc>GFP* is stained with GFP and Ance. A series of sections from basal to apical regions show no ectopic expression of Ance. Scale bar, 50 µm

(b) Wing disc expressing GFP and Tkv^{QD} shows ectopic Ance protein indicated by arrows. Serial sections show that ectopic Ance expression is localized preferentially to the proximal regions of the *ptc* stripe. Scale bar, 50 µm

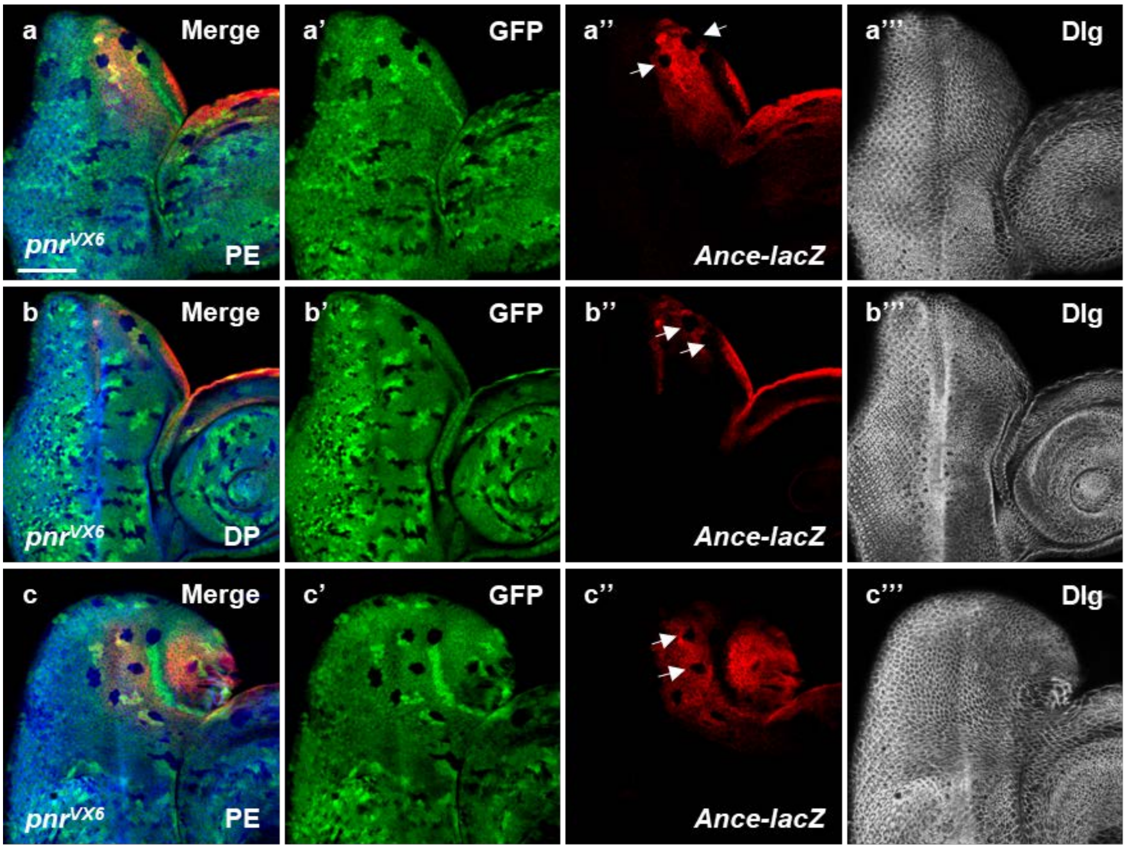

Fig. S6. *pnr* is required for *Ance-lacZ* expression

pnr^{VX6} mutant clones result in reduction of *Ance-lacZ* expression in the dorsal eye disc (arrows). (a) Eye disc at the PE level. (b) Disc proper. (c) Another eye disc with *pnr* mutant clones shown at the PE level (arrows). *Ance-lacZ* is lost in *pnr* mutant clones. (a, b, c) Merge, (a’, b’, c’) GFP, (a’’, b”, c’’) *Ance-lacZ*, (a’’’, b”’, c’’’) Dlg. Scale bar, 50 µm

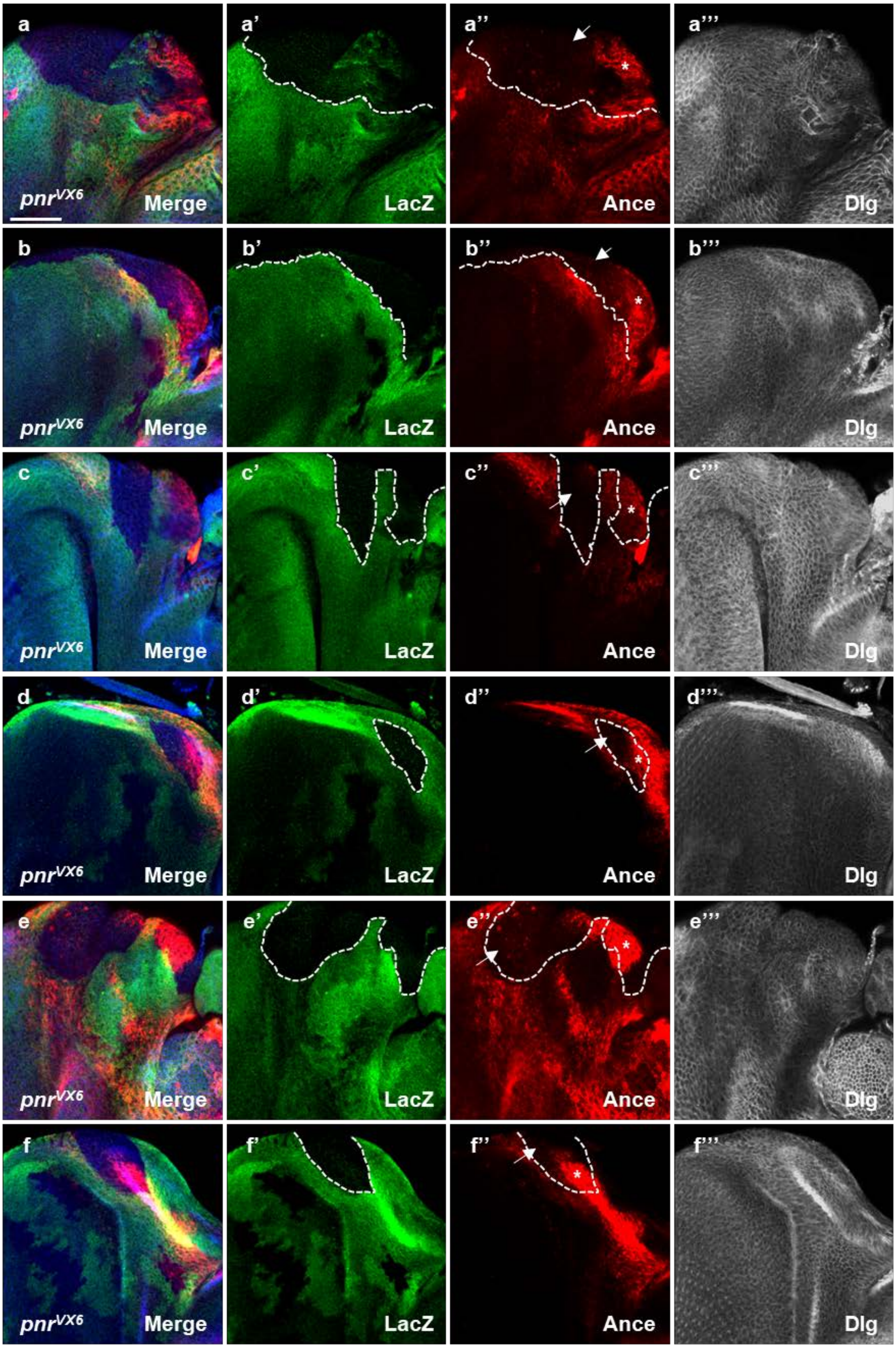
a
pnrVX6
Merge
a'
LacZ
a''
Ance
a'''
Dlg
b
pnrVX6
Merge
b'
LacZ
b''
Ance
b'''
Dlg
c
pnrVX6
Merge
c'
LacZ
c''
Ance
c'''
Dlg
d
pnrVX6
Merge
d'
LacZ
d''
Ance
d'''
Dlg
e
pnrVX6
Merge
e'
LacZ
e''
Ance
e'''
Dlg
f
pnrVX6
Merge
f'
LacZ
f''
Ance
f'''
Dlg

Fig. S7. *pnr* is required for Ance expression.

pnr^{VX6} mutant clone (marked by the absence of LacZ staining) reduces Ance protein level in the dorsal eye disc. A large mutant clones show considerable reduction in Ance staining as indicated by arrow. In some areas, Ance proteins still remain in pnr^{VX6} clones as indicated by asterisk. Other pnr^{VX6} mutant clones showing loss or variable Ance levelsare shown (a) to (f).

(a-f) Merge, (a'-f') LacZ, (a''-f'') Ance, (a'''-f''') Dlg (blue channel in black/white). Scale bar, 50 μm

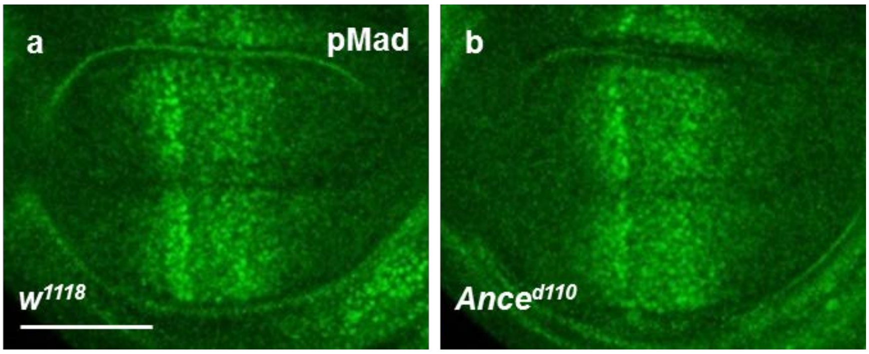

Fig. S8. Loss of Ance does not affect the level of phosphorylated Mad.

(a) Phospho-Mad (pMad) staining in w^{1118} wing disc. Wing pouch of w^{1118} shows a graded pMad staining pattern along the anterior posterior axis.

(b) pMad staining in $Ance^{d110}$ wing disc. Wing pouch of $Ance^{d110}$ shows a graded pMad staining pattern. There is no significant difference of pMad expression between w^{1118} and $Ance^{d110}$. Scale bars, 50µm.

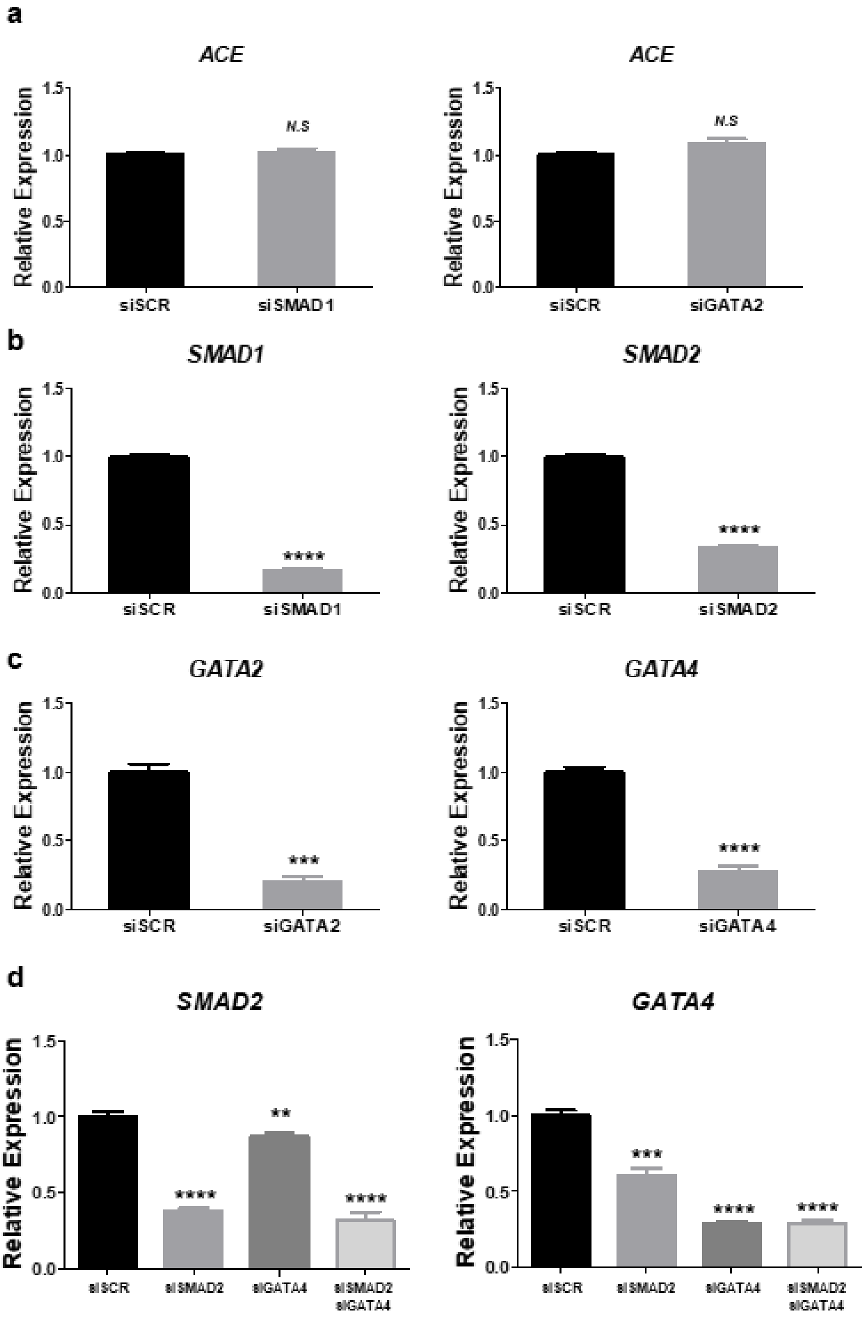

Fig. S9. *ACE* expression under *SMAD1* or *GATA2* siRNA treatment and knockdown efficiency of *SMAD1, SMAD2, GATA2,* and *GATA4* siRNAs in HEK293 cells.

(a) siRNA for *SMAD1* or *SMAD2* leads to reduction in *SMAD1* or *SMAD2* expression in HEK293 cells. Each siRNA against *SMAD1* and *SMAD2* leads to reduction of *SMAD1* (16.3%)

and *SMAD2* (33.6%), respectively.

(b) siRNA for *SMAD1* or *SMAD2* leads to reduction in *SMAD1* or *SMAD2* expression in HEK293 cells. Each siRNA against *SMAD1* and *SMAD2* leads to reduction of *SMAD1* (16.3%) and *SMAD2* (33.6%), respectively.

(c) siRNA for *GATA2* or *GATA4* leads to reduction in *GATA2* or *GATA4* expression in HEK293 cells. Each siRNA against *GATA2* and *GATA4* leads to reduction of *GATA2* (26.1%) and *GATA4* (27.7%), respectively.

(d) Downregulated *SMAD2* and *GATA4* levels in double siRNAs for *SMAD2* and *GATA4* in HEK293 cells. *SMAD2* siRNA leads to reduced *SMAD2* expression (38.3%). *GATA4* siRNA leads to reduced *SMAD2* expression (86.7%). Double siRNAs for *GATA4* and *SMAD2* result in reduced *SMAD2* reduction (31.6%). *SMAD2* siRNA leads to reduced *GATA4* expression (60.5%). *GATA4* siRNA leads to reduced *GATA4* expression (28.9%). Double siRNAs for *GATA4* and *SMAD2* result in reduced *GATA4* reduction (28.1%). All data represent the mean±s.e.m. (error bars) and *P*-values were calculated by using Student's *t*-test. ***P* <0.01, ****P* <0.001, *****P* <0.0001.

Table S1. siRNA sequence for HEK293 cells.

Gene symbol	siRNA Sequence
SMAD1	CCCAAUAGCAGUUACCCAA
SMAD2	GCAGAACUA UCUCCUACUA
GATA2	GAAGGGAUCCAGACUCGGA
GATA4	UCAGUAUUUAACUAAUAAA

Table S2. RT-PCR and qRT-PCR primer.

Gene symbol		Sequence
Ance	Forward	5'- TCATCACGAACTGGGACACA -3'
	Reverse	5'- CTCGTCATCGCGCACATAAT-3'
Rp49	Forward	5'- TACAGGCCCAAGATCGTGAA -3'
	Reverse	5'- TCTCCTTGCGCTTCTTGGA -3'
ACE	Forward	5'- TAGCCCTCTCAGTGTCTACG -3'
	Reverse	5'- CGAGGTAGCTGAAGGGGATA -3'
SMAD1	Forward	5'- GCAACCGAGTAACTGTGTCA -3'
	Reverse	5'- AGGAGGAAGTACAGGGCTTT -3'
SMAD2	Forward	5'- CCTTCCTCAACCTTTGCTGT -3'
	Reverse	5'- GCTCACAAGATGGGTAGTGG -3'
GATA2	Forward	5'- CCTCCAGCTTCACCCCTAA -3'
	Reverse	5'- CACAGGCATTGCACAGGTAGT -3'
GATA4	Forward	5'- TCTGCCTGGTAATGACTCCA -3'
	Reverse	5'- GCGTGTAAAGGCATCTGAGA -3'
HPRT	Forward	5'- TGACCTTGATTTATTTTGCATACC -3'
	Reverse	5'- CGAGCAAGACGTTCAGTCCT -3'

Table S3. qRT-PCR primer in ChIP assays.

Gene symbol		Sequence
Primer 1	Forward	5'- GGAAATGCCTTGGAGTCAGA -3'
	Reverse	5'- GAAGAGCCTGAGGCCTAACC -3'
Primer 2	Forward	5'- AACTTTTCTGCTGACGAGGC -3'
	Reverse	5'- CAACATCACCGCGGAGAAT -3'
Primer 3	Forward	5'- GCAGAGCAGCTGGTATGACA -3'
	Reverse	5'- GAGGCAAAGAGGAGCATCAG -3'

Figure 3d

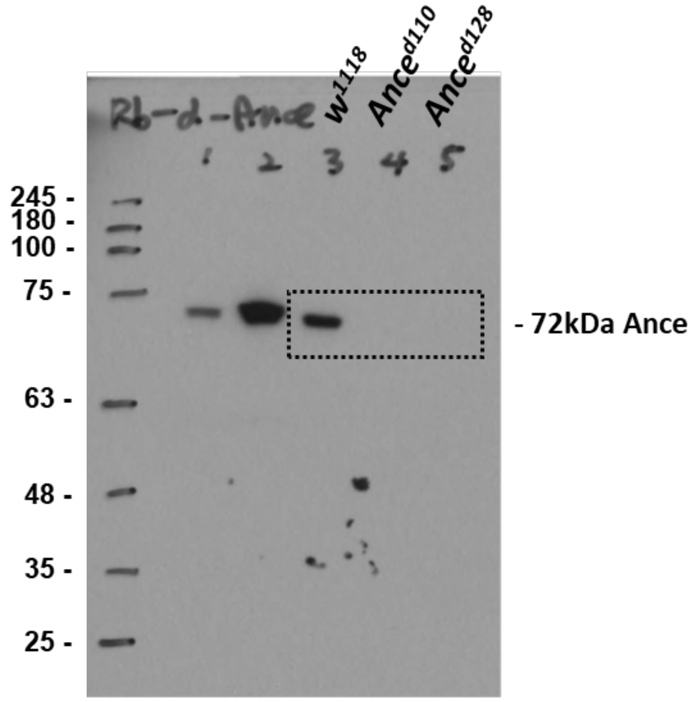

Rb-α-Ance
w^{1118}
$Ance^{d110}$
$Ance^{d128}$
1 2 3 4 5
245 -
180 -
100 -
75 -
63 -
48 -
35 -
25 -
- 72kDa Ance

Figure 5c

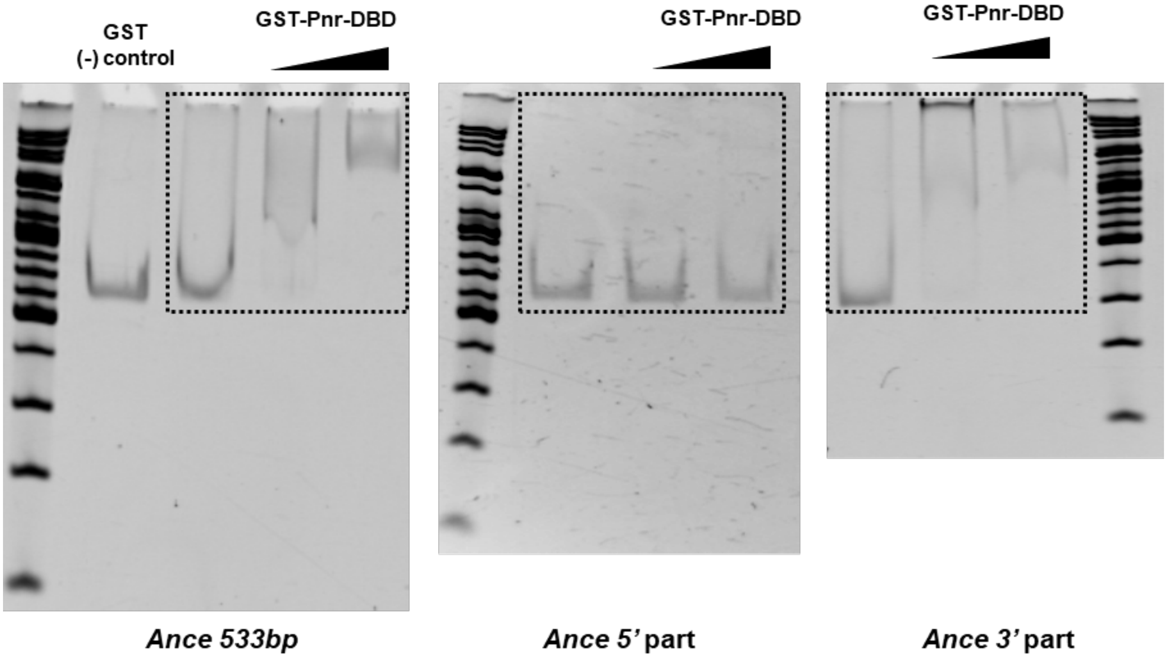
GST
(-) control
GST-Pnr-DBD
GST-Pnr-DBD
GST-Pnr-DBD
Ance 533bp
Ance 5' part
Ance 3' part

Figure 6e

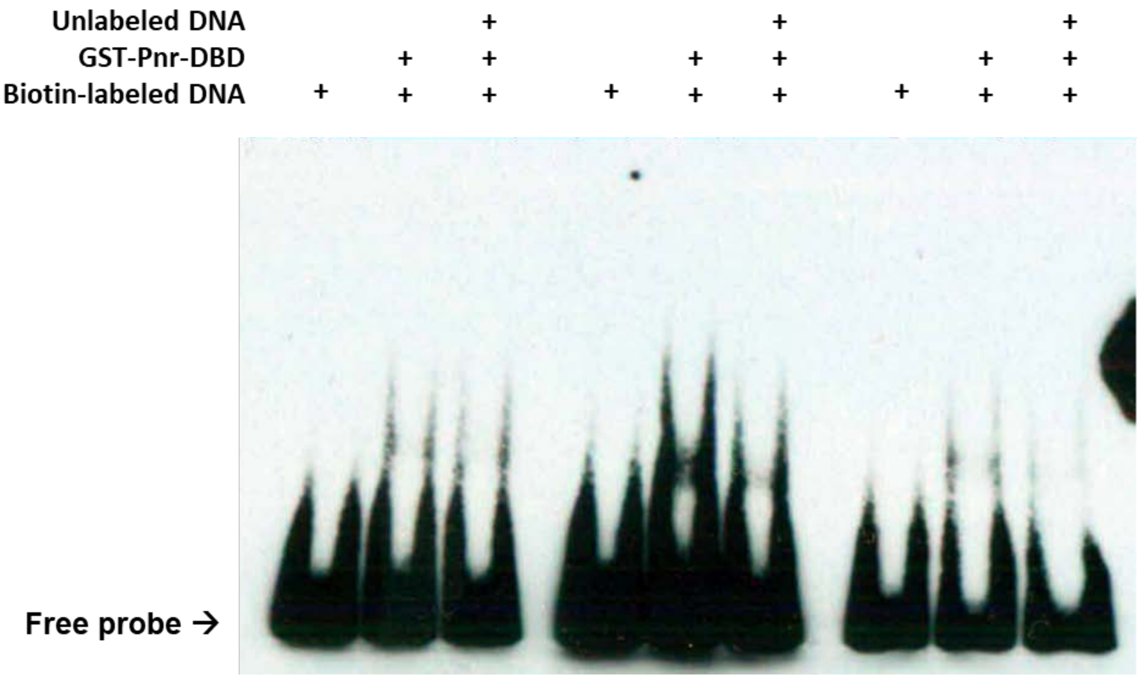

Figure 6f

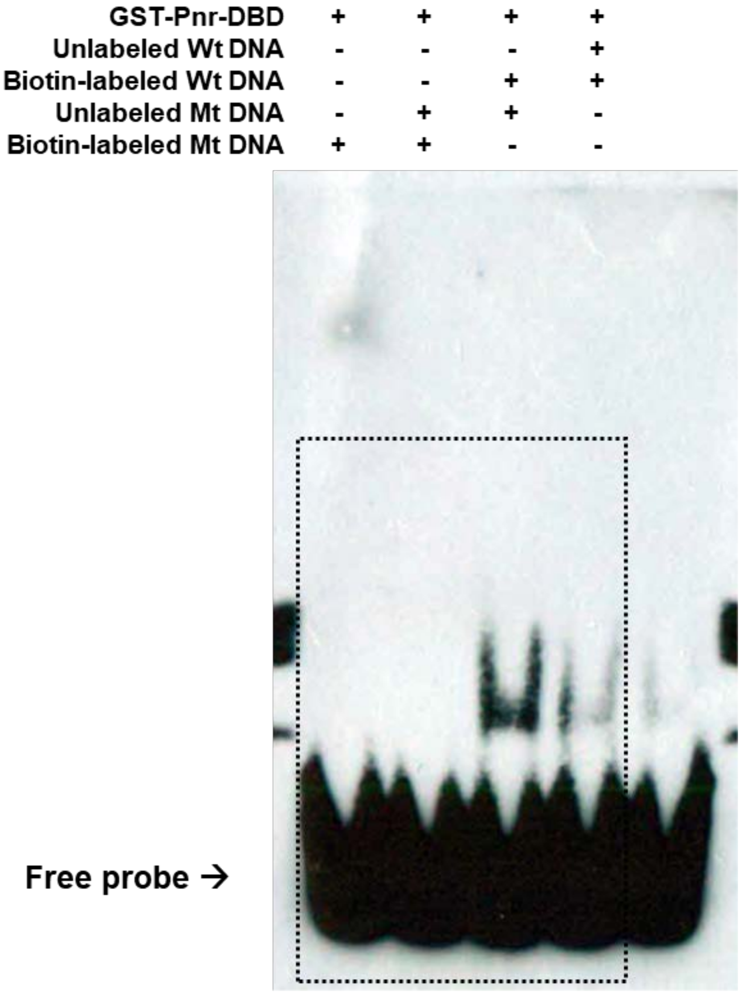

GST-Pnr-DBD + + + +
Unlabeled Wt DNA - - - +
Biotin-labeled Wt DNA - - + +
Unlabeled Mt DNA - + + -
Biotin-labeled Mt DNA + + - -
Free probe →

Figure 7e

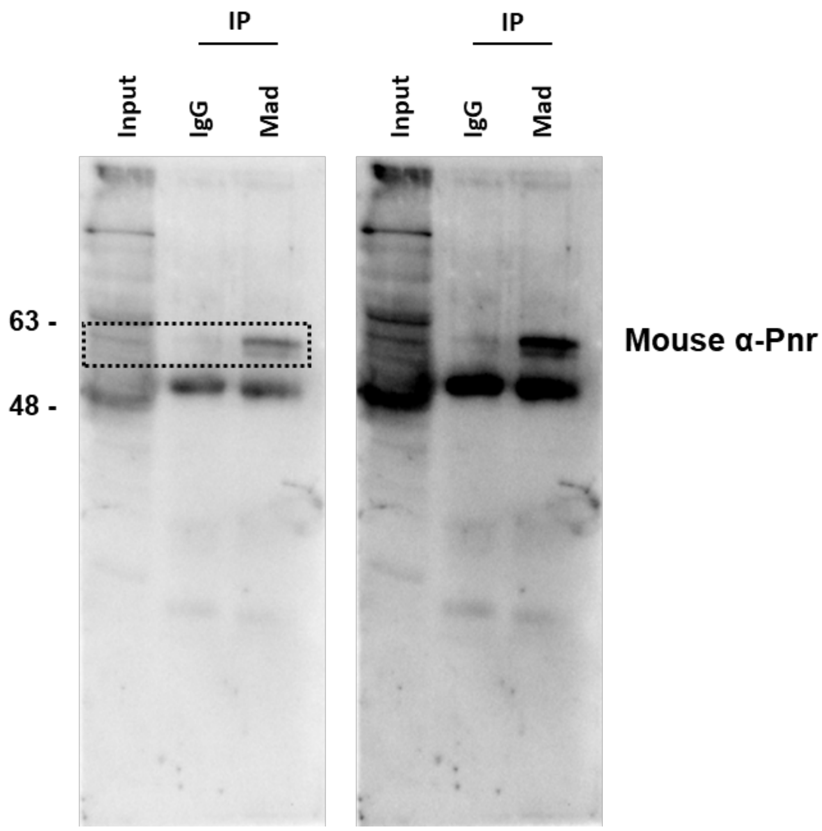

Figure 7f

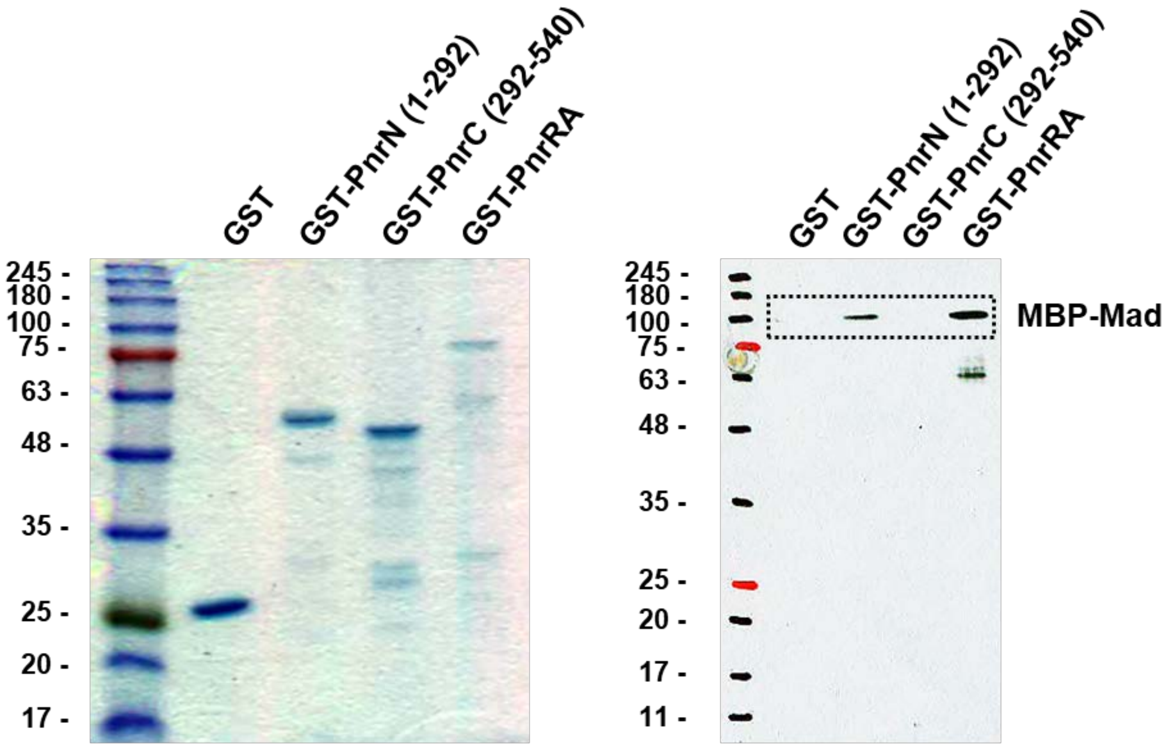
GST
GST-PnrN (1-292)
GST-PnrC (292-540)
GST-PnrRA
245 -
180 -
100 -
75 -
63 -
48 -
35 -
25 -
20 -
17 -
GST
GST-PnrN (1-292)
GST-PnrC (292-540)
GST-PnrRA
245 -
180 -
100 -
75 -
63 -
48 -
35 -
25 -
20 -
17 -
11 -
MBP-Mad

Figure 8f

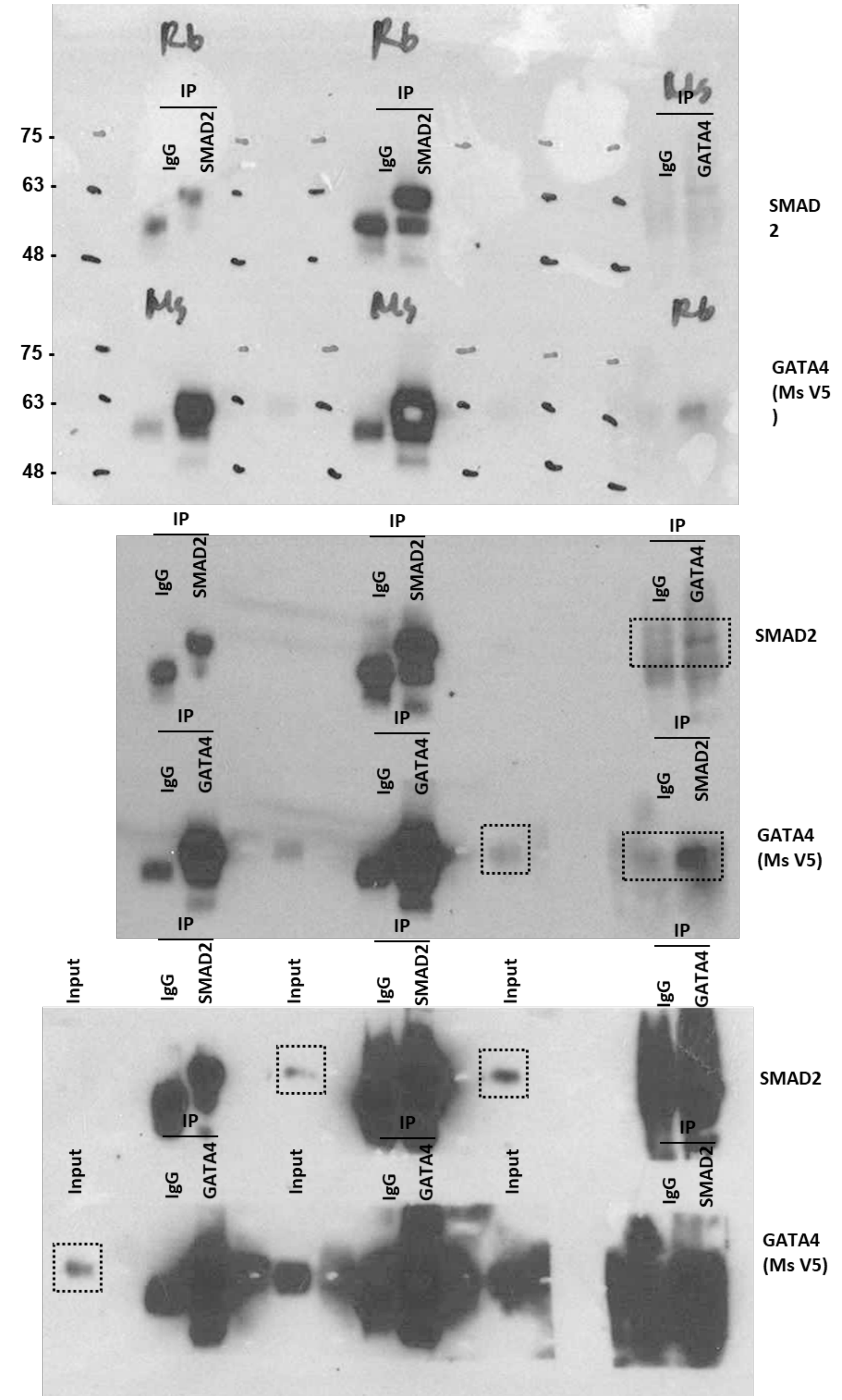

Rb
Rb
Ms
IP
IP
IP
IgG
SMAD2
IgG
SMAD2
IgG
GATA4
75
63
48
SMAD 2
Ms
Ms
Rb
75
63
48
GATA4 (Ms V5)
SMAD2
GATA4 (Ms V5)
Input
SMAD2
GATA4 (Ms V5)

Figure 8g, 8h

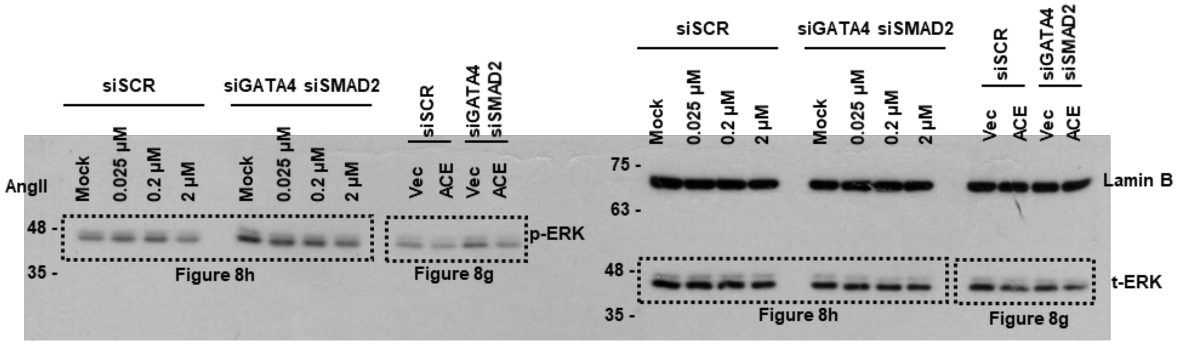
siSCR
siGATA4 siSMAD2
siSCR
siGATA4 siSMAD2
AngII
Mock
0.025 μM
0.2 μM
2 μM
Vec
ACE
48 -
35 -
p-ERK
Figure 8h
Figure 8g
75 -
63 -
Lamin B
t-ERK
